# Plasma‐Assisted Defect Engineering on *p‐n* Heterojunction for High‐Efficiency Electrochemical Ammonia Synthesis

**DOI:** 10.1002/advs.202205786

**Published:** 2023-01-22

**Authors:** Jiameng Liu, Linghao He, Shuangrun Zhao, Sizhuan Li, Lijun Hu, Jia‐Yue Tian, Junwei Ding, Zhihong Zhang, Miao Du

**Affiliations:** ^1^ College of Material and Chemical Engineering Institute of New Energy Science and Technology School of Future Hydrogen Energy Technology Zhengzhou University of Light Industry Zhengzhou 450001 P. R. China

**Keywords:** boron nanosheets, defect engineering, electrocatalytic nitrogen reduction reaction (eNRR), p‐n heterojunctions, semiconductive metal–organic frameworks

## Abstract

A defect‐rich 2D *p‐n* heterojunction, Co*_x_* Ni_3‐_ *_x_* (HITP)_2_/BNSs‐P (HITP: 2,3,6,7,10,11‐hexaiminotriphenylene), is constructed using a semiconductive metal–organic framework (MOF) and boron nanosheets (BNSs) by in situ solution plasma modification. The heterojunction is an effective catalyst for the electrocatalytic nitrogen reduction reaction (eNRR) under ambient conditions. Interface engineering and plasma‐assisted defects on the *p‐n* Co_x_Ni_3‐x_(HITP)_2_/BNSs‐P heterojunction led to the formation of both Co‐N_3_ and B…O dual‐active sites. As a result, Co*_x_* Ni_3‐x_(HITP)_2_/BNSs‐P has a high NH_3_ yield of 128.26 ± 2.27 µg h^−1^ mg_cat._^−1^ and a Faradaic efficiency of 52.92 ± 1.83% in 0.1 m HCl solution. The catalytic mechanism for the eNRR is also studied by in situ FTIR spectra and DFT calculations. A Co*_x_* Ni_3‐_ *_x_* (HITP)_2_/BNSs‐P‐based Zn‐N_2_ battery achieved an unprecedented power output with a peak power density of 5.40 mW cm^−2^ and an energy density of 240 mA h g_zn_^−1^ in 0.1 m HCl. This study establishes an efficient strategy for the rational design, using defect and interfacial engineering, of advanced eNRR catalysts for ammonia synthesis under ambient conditions.

## Graphical abstract

A new type of *p*‐*n* type heterojunction is constructed using a semiconductive MOF and boron nanosheets (BNSs) through in situ solution plasma modification. Interface and plasma‐assisted defect engineering endow the heterojunction with multiple active sites, abundant defects, and modulated electronic structures, which show outstanding performance for electrocatalytic ammonia synthesis using a Zn‐N_2_ battery.

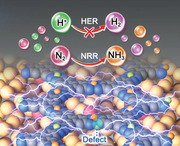

## 1 Introduction

Ammonia (NH_3_) is critically important to the functioning of modern civilization and has widespread applications in the chemical industry, agriculture, and medicine.^[^ ^1^ ^]^ Electrocatalytic synthesis can convert ubiquitous N_2_ and H_2_O to NH_3_ under ambient conditions and is an alternative to the Haber–Bosch process that operates at high temperatures and pressures using highly purified H_2_/N_2_ gas.^[^ ^2^ ^]^ Unfortunately, electrocatalysts for the electrolytic nitrogen reduction reaction (eNRR) have poor NH_3_ yields and Faradaic efficiencies (FEs), primarily caused by the low density of active sites and the competing hydrogen evolution reaction (HER).^[^ ^3^ ^]^ Thus, different approaches, such as defect engineering, interface engineering, heteroatom doping, and single metal catalysis, have improved eNRR performance.^[^ ^4^ ^]^ Among these, interfacial engineering is effective owing to the high‐speed electron transport channels and electronic coupling effect between different components, which accelerates electron transfer and tunes the free energies of the reaction intermediates.^[^ ^5^ ^]^ The electronic structures reach a thermal equilibrium state for a *p*‐*n* junction formed by *p*‐type and *n*‐type semiconductors, generating the opposite space‐charge region at the interface.^[^ ^6^ ^]^ The interfacial charge‐transfer efficiency in the built‐in electric field of a *p*‐*n* heterojunction can be increased, and more charge can be stored. Modulating the electronic structure will greatly influence the absorption of the target species and charge transfer during electrochemical reactions.^[^ ^7^ ^]^

Metal–organic frameworks (MOFs) possess tailorable structures, high porosity, and well‐defined pores,^[^ ^8^ ^]^ and show great promise as catalysts of electrocatalytic reactions.^[^ ^9^ ^]^ In addition to their inherent properties, MOFs can be further functionalized through interactions with other active components.^[^ ^10^ ^]^ Moreover, the coordination bonds in MOFs can be readily broken to introduce different defects into pristine MOF structures.^[^ ^11^ ^]^ These defects can act as Lewis acid sites to greatly enhance catalytic activity and extract *π* electrons from N_2_ molecules, reducing the N≡N decomposition energy and facilitating eNRR efficiency.^[^ ^12^ ^]^ Nonetheless, MOFs often have poor eNRR activity owing to their intrinsically low electrical conductivities. In this regard, conductive or semiconductive MOFs constructed using *π*‐conjugated aromatic ligands^[^ ^13^ ^]^ exhibit improved electrocatalytic performance^[^ ^14^ ^]^ and possible applications in the eNRR.^[^ ^15^ ^]^ For instance, theoretical calculations reveal that a 2D Mo‐based conductive MOF may be used as the eNRR catalyst with a low overpotential of 0.18 V.^[^ ^16^ ^]^ A 2D Co‐based conductive MOF Co_3_(HHTP)_2_ has an NH_3_ yield of 22.14 µg h^−1^ mg_cat._^−1^ and FE of 3.34% at −0.40 V.^[^ ^17^ ^]^

Notably, 2D boron nanosheets (BNSs), have exceptional mechanical, physical, and chemical properties.^[^ ^18^ ^]^ These can fix and activate N_2_ molecules because the B atoms in 2D boron sheets are electron‐deficient.^[^ ^5c^ ^]^ However, pristine BNSs have limited application as eNRR catalysts because although they activate N_2_ and reduce it to NH_3_, the latter is not released properly.^[^ ^18^ ^]^ Furthermore, they have poor stability^[^ ^19^ ^]^ and few active sites. Considering the advantages of semiconducting MOFs and BNSs, a promising approach may be to develop heterostructured eNRR catalysts based on these two components via interface engineering. As far as we are aware, this idea has not yet been explored.

To this end, we have prepared a unique *p‐n* heterojunction of semiconductive MOF Co_x_Ni_3‐x_(HITP)_2_ and BNSs, denoted as Co_x_Ni_3‐x_(HITP)_2_/BNSs‐P, by in situ solution plasma modification. The generation of an effective heterojunction at the interface between the ultrathin MOF and BNSs under plasma treatment can significantly lower the work function of the catalyst and decrease the active energy of N_2_ molecules. The solution plasma technique can be utilized to generate many vacancies and defects in the catalysts.^[^ ^20^ ^]^ Introducing a second metal ion into MOFs skeletons can generate more unsaturated metal‐coordination sites, thereby modulating the electron density of the active sites and improving catalytic activity.^[^ ^21^ ^]^ Thus, Co_x_Ni_3‐x_(HITP)_2_/BNSs‐P had a high ammonia yield of 128.26 ± 2.27 µg h^−1^ mg_cat._^−1^ and FE of 52.92 ± 1.83% versus a reversible hydrogen electrode (RHE) in 0.1 m HCl solution, which greatly exceeds the performance of the individual components and previously reported catalysts. Moreover, the Co*_x_* Ni_3‐_ *_x_* (HITP)_2_/BNSs‐P‐based aqueous Zn‐N_2_ battery achieves a high NH_3_ yield of 15.48 µg h^−1^ mg_cat._^−1^ and a large power density of 5.40 mW cm^−2^, which shows promising potential for NH_3_ production.

## 2 Results and Discussion

**Figure 1:**
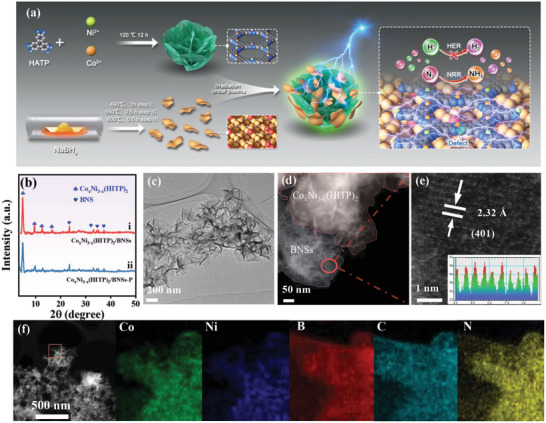
a) Schematic illustration of the fabrication process of Co_x_Ni_3‐x_(HITP)_2_/BNSs‐P for eNRR. b) PXRD patterns of i) Co_x_Ni_3‐x_(HITP)_2_/BNSs and ii) Co_x_Ni_3‐x_(HITP)_2_/BNSs‐P. c) TEM image, d,e) HAADF‐STEM image (inset: lattice line scanning), and f) elemental mapping of Co (green), Ni (blue), B (red), C (cyan‐blue), and N (yellow) in Co_x_Ni_3‐x_(HITP)_2_/BNSs‐P.

The Co*_x_* Ni_3‐_ *_x_* (HITP)_2_/BNSs‐P heterojunction was prepared by solution plasma treatment of a suspension containing BNSs and Co*_x_* Ni_3‐_ *_x_* (HITP)_2_ at an input power of 200 W (**Figure** **1**a). The Mott–Schottky (MS) analysis reveals (Figure S1a,b, Supporting Information) Co*_x_* Ni_3‐_ *_x_* (HITP)_2_‐P and BNSs‐P the negative and positive slopes, corresponding to *p*‐type and *n*‐type semiconductors, respectively. The normalized secondary electron cutoff energy, measured by ultraviolet photoelectron spectroscopy (Figure S2a, Supporting Information), illustrates the work function (*Φ*) of the electrocatalysts. The results (Figure S2b, Supporting Information) reveal that Co*_x_* Ni_3‐_ *_x_* (HITP)_2_/BNSs‐P exhibits a smaller *Φ* (2.27 eV) than those of the individual components. This observation suggests that strong electronic coupling can occur at the interface of the two components, facilitating charge mediation and promoting the eNRR.

The powder X‐ray diffraction (PXRD) pattern of Co*_x_* Ni_3‐_ *_x_* (HITP)_2_/BNSs (Figure 1b, curve *i*) clearly integrates the characteristic peaks of Co*_x_* Ni_3‐_ *_x_* (HITP)_2_ (Figure S5a, curve *i*, Supporting Information) and BNSs (Figure S5b, curve *i*, Supporting Information). Furthermore, plasma modification did not change the crystal structures, as no significant variation is found in the PXRD patterns (Figure 1b and Figure S6a,b, Supporting Information). Moreover, the field emission scanning electron microscopy (FE‐SEM) images of the samples before and after plasma treatment are very similar and comprise a large number of ultrathin NSs (Figure S7a,b, Supporting Information). This result is confirmed by transmission electron microscopy (TEM) images (Figure 1c and Figure S8c,d, Supporting Information). It is clear that the Co*_x_* Ni_3‐_ *_x_* (HITP)_2_ NSs are in close contact with the BNSs (Figure 1d). The high‐angle annular dark‐field STEM (HAADF‐STEM) image (Figure 1d) shows the connected structure of the two components. The reconstructed image (inset of Figure 1e) has a lattice spacing of 2.32 Å, corresponding to the (401) plane of the boron sheet.^[^ ^22^ ^]^ The energy‐dispersive X‐ray spectroscopy (EDS) images of Co*_x_* Ni_3‐_ *_x_* (HITP)_2_/BNSs before and after plasma treatment (Figure 1f and Figure S7e, Supporting Information) indicate that all elements were homogeneously dispersed throughout the selected region. Additionally, the surface morphologies of BNS and Co_x_Ni_3‐x_(HITP)_2_ before and after plasma modification (Figures S8 and S9, Supporting Information) reveal no apparent change. The mass amounts of Co and Ni in Co*_x_* Ni_3‐_ *_x_* (HITP)_2_/BNSs were 3.96 wt.% and 5.36 wt.%, respectively, smaller than those of Co*_x_* Ni_3‐_ *_x_* (HITP)_2_ (Co:5.27 wt.%; Ni:7.12 wt.%), as determined by inductively coupled plasma‐mass spectrometry. In addition, the Co*_x_* Ni_3‐_ *_x_* (HITP)_2_/BNSs junction exhibits a smaller Brunauer—Emmett–Teller (BET) surface area of 368.2 m^2^ g^−1^ than Co*_x_* Ni_3‐_ *_x_* (HITP)_2_ (398.7 m^2^ g^−1^) due to the introduction of BNSs (Figure S12 and Table S1, Supporting Information).

**Figure 2:**
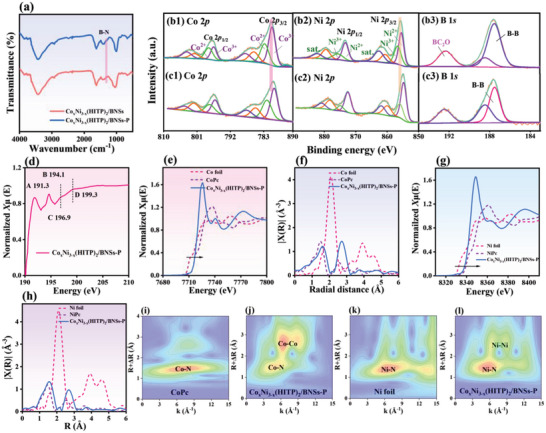
a) FTIR spectra of Co_x_Ni_3‐x_(HITP)_2_/BNSs and Co_x_Ni_3‐x_(HITP)_2_/BNSs‐P. Co 2*p*, Ni 2*p*, and B 1*s* XPS spectra of b) Co_x_Ni_3‐x_(HITP)_2_/BNSs and c) Co_x_Ni_3‐x_(HITP)_2_/BNSs‐P. d) B K‐edge XANES, e) Co *K*‐edge XANES, and f) FT‐EXAFS spectra of Co_x_Ni_3‐x_(HITP)_2_/BNSs‐P. g) Ni K‐edge XANES and h) corresponding FT‐EXAFS spectra of Co_x_Ni_3‐x_(HITP)_2_/BNSs‐P. WT‐EXAFS contour maps of i) CoPc, k) NiPc, and j,l) Co_x_Ni_3‐x_(HITP)_2_/BNSs‐P.

The Fourier transform infrared (FTIR) spectra of Co_x_Ni_3‐x_(HITP)_2_/BNSs (**Figure** **2**a) show characteristic peaks at 1043 and 3433 cm^−1^ due to the stretching and in‐plane bending of ‐NH in secondary amines, respectively.^[^ ^23^ ^]^ However, a new characteristic peak at ≈1306 cm^−1^ found for Co_x_Ni_3‐x_(HITP)_2_/BNSs‐P is assigned to B–N vibration. This weak B–N vibration is also observed in the FTIR spectrum of BNSs‐P, whereas no change is observed in the FTIR spectra of Co*_x_* Ni_3‐_ *_x_* (HITP)_2_ before and after plasma treatment (Figure S13a,b, Supporting Information).

The electron effect at the interface of BNSs and Co*_x_* Ni_3‐_ *_x_* (HITP)_2_ was characterized by X‐ray photoelectron spectroscopy (XPS). The Co 2*p* and Ni 2*p* XPS spectra of Co*_x_* Ni_3‐_ *_x_* (HITP)_2_/BNSs and Co_x_Ni_3‐x_(HITP)_2_/BNSs‐P (Figure 2b1,b2,c1,c2) reveal the coexistence of mixed Co^3+^/Co^2+^ and Ni^3+^/Ni^2+^ species. Both Co^2+^ 2*p*_3/2_ and Ni^2+^ 2*p*_3/2_ species in Co_x_Ni_3‐x_(HITP)_2_/BNSs‐P show a negative shift in the binding energy (Figure 2c1,2) in comparison with those of Co_x_Ni_3‐x_(HITP)_2_/BNSs, suggesting electron transfer from BNSs to Co*_x_* Ni_3‐_ *_x_* (HITP)_2_. The B 1*s* spectrum of the junction (Figure 2b3,c3) can be divided into B–B (187.6 eV), B‐B (188.2 eV), and BC_2_O (192.3 eV).^[^ ^24^ ^]^ The coexistence of B–O and BC_2_O suggests that carbon and oxygen atoms are introduced into the six‐membered ring B12 units of the BNSs. This could be due to the oxidation of BNSs when exposed to air; this surface passivation affords boron sheets with good stability.^[^ ^25^ ^]^ As a result, the relative content of B‐OH in the B 1*s* spectrum for Co*_x_* Ni_3‐_ *_x_* (HITP)_2_/BNSs‐P (≈26%) is higher than that of Co*_x_* Ni_3‐_ *_x_* (HITP)_2_/BNSs (≈20%). Plasma exposure can decompose water into reactive species such as free radicals (O•, O•^–^ or O_2_•^–^), positive ions (O^+^ and O^2+^), negative ions (O^−^ and O^2−^), and electrons in the reaction channel,^[^ ^17^ ^]^ while simultaneously activating the boron. The O‐related species originating from water can react with activated B to form a B–O bond. The increased B–OH functionality can increase stability and enhance the adsorption and activation of N_2_.^[^ ^26^ ^]^ The N 1*s* XPS spectrum of Co_x_Ni_3‐x_(HITP)_2_/BNSs exhibits pyridinic N (398.8 eV), pyrrolic N (399.7 eV), graphitic N (400.7 eV), and oxidized N (401.9 eV) (Figure S17a2, Supporting Information). However, the N 1*s* of Co_x_Ni_3‐x_(HITP)_2_/BNSs‐P (Figure S17b2, Supporting Information) shifts negatively by 0.17 eV due to the production of N defects during the plasma treatment.^[^ ^27^ ^]^ The graphene‐like Co*_x_* Ni_3‐_ *_x_* (HITP)_2_ and Co*_x_* Ni_3‐_ *_x_* (HITP)_2_/BNSs were not affected by the plasma treatment, as confirmed by the Raman spectra (Figure S18a,b,c, Supporting Information).

Electron paramagnetic resonance (EPR) spectra were obtained to elucidate the defect formation mechanism. The signals at g = 2.003 in the EPR spectra of BNSs and BNSs‐P (Figure S19a, Supporting Information) reveal the presence of oxygen vacancies, where the increased peak intensity of BNSs‐P indicates additional oxygen vacancies caused by the plasma modification. Similarly, the EPR spectra of Co*_x_* Ni_3‐_ *_x_* (HITP)_2_ and Co*_x_* Ni_3‐_ *_x_* (HITP)_2_‐P (Figure S19b, Supporting Information) show a signal at *g* = 2.046 due to the HITP‐centered radical.^[^ ^28^ ^]^ The enhanced signal of Co*_x_* Ni_3‐_ *_x_* (HITP)_2_‐P suggests the formation of unsaturated metal coordination. The signal intensity of Co*_x_* Ni_3‐_ *_x_* (HITP)_2_/BNSs‐P was enhanced compared to that of Co*_x_* Ni_3‐_ *_x_* (HITP)_2_/BNSs, suggesting the formation of an increased number of defects. The number of defects was also higher than in Co*_x_* Ni_3‐_ *_x_* (HITP)_2_ because of the variation in the electronic structure of the metal sites in the heterojunction. As a result, the Co*_x_* Ni_3‐_ *_x_* (HITP)_2_/BNSs‐p heterostructure includes two types of defects, namely oxygen vacancies and unsaturated coordination, which synergistically enhance its eNRR activity.^[^ ^11^ ^]^ Thermogravimetric analysis tests of Co*_x_* Ni_3‐_ *_x_* (HITP)_2_ and Co*_x_* Ni_3‐x_(HITP)_2_/BNSs before and after the plasma treatment were carried out under an N_2_ atmosphere (Figure S20, Supporting Information). The decomposition temperature of Co*_x_* Ni_3‐_ *_x_* (HITP)_2_‐P was ≈187 °C, significantly lower than that of Co*_x_* Ni_3‐_ *_x_* (HITP)_2_ (276 °C). It indicates the enhanced thermal stability of Co*_x_* Ni_3‐_ *_x_* (HITP)_2_‐P, which may have been caused by the presence of N defects. A significant mass loss (≈18 wt.%) is observed within a temperature range of 187–470 °C for Co*_x_* Ni_3‐_ *_x_* (HITP)_2_‐P. When the temperature is higher than 900 °C, the mass loss of Co*_x_* Ni_3‐_ *_x_* (HITP)_2_‐P is up to 44 wt.%, smaller than that of Co*_x_* Ni_3‐_ *_x_* (HITP)_2_ (53 wt.%). Both Co*_x_* Ni_3‐_ *_x_* (HITP)_2_/BNSs and Co*_x_* Ni_3‐_ *_x_* (HITP)_2_/BNSs‐P show similar thermal decomposition, suggesting their comparable thermal stabilities. This finding suggests that the incorporation of BNSs can accelerate structural stability owing to the presence of numerous defects.

The metal coordination structure of Co_x_Ni_3‐x_(HITP)_2_/BNSs‐P was studied by obtaining X‐ray absorption near edge structure (XANES) and extended X‐ray absorption fine structure (EXAFS) spectra. The *K*‐edge spectrum of B in Co_x_Ni_3‐x_(HITP)_2_/BNSs‐P (Figure 2d) consisted of four resonances with peak centers of 191.3 (peak A), 194.1 (peak B), 196.9 (peak C), and 199.3 eV (peak D). The A and C peaks revealed the trigonal coordination of BNSs, and the sharp and strong peak B at 194.1 eV is attributed to the B–O π* resonance. The larger separation between the C and D peaks indicated that the distance between adjacent B atoms was relatively small. The XANES spectrum of the Co absorption edge of Co_x_Ni_3‐x_(HITP)_2_/BNSs‐P (Figure 2e) shifted to a higher energy than that of the reference Co foil but lower than that of CoO. Thus, the average valences of the Co species were located in Co(0) and Co(II). Moreover, the Co valence in Co_x_Ni_3‐x_(HITP)_2_/BNSs‐P was similar to that of Co‐phthalocyanine (CoPc), owing to the low electronegativity of N compared to O. This verified partial charge transfer from N to Co, illustrating that Co was successfully coordinated to HITP. The Fourier transform (FT)‐EXAFS spectrum of Co_x_Ni_3‐x_(HITP)_2_/BNSs‐P (Figure 2f) included Co‐N (1.56 Å) and Co‐Ni/Co (2.70 Å) peaks. The Co–Co path is the distance between Co atoms in adjacent layers, revealing weak metal‐metal (d–d) interactions and strong *π*–*π* interactions between adjacent layers. The interatomic distance (R) and coordination number (CN) of each shell in Co_x_Ni_3‐x_(HITP)_2_/BNSs‐P (Figure S21 and Table S2, Supporting Information) show a coordination number of 3.38 for Co–N. This indicates that the Co atom in Co_x_Ni_3‐x_(HITP)_2_ is coordinately unsaturated, affording high Lewis acidity. The empty *d*‐orbital of metallic Co can capture the lone electron pair of the Lewis base (N_2_ molecule), thereby accelerating the eNRR kinetics and suppressing the HER.^[^ ^29^ ^]^ Wavelet transform (WT) analysis of Co_x_Ni_3‐x_(HITP)_2_/BNSs‐P reveals maximum intensities at 4.02 and 5.73 Å^−1^ for the Co–N and Co–Co paths, respectively (Figure 2i,j). The maximum *k*‐value of the Co‐Co path in Co_x_Ni_3‐x_(HITP)_2_/BNSs‐P was larger than that of Co‐Co in CoO (5.45 Å^−1^) (Figure S21a, Supporting Information). The absorption edge of Ni in Co_x_Ni_3‐x_(HITP)_2_/BNSs‐P is between those of the Ni foil and NiO, similar to that of Ni‐phthalocyanine (NiPc), indicating that the average valence state of Ni is between that of Ni(0) and Ni(II) (Figure 2g). Also, the Ni *K*‐edge of Co_x_Ni_3‐x_(HITP)_2_/BNSs‐P shows two peaks at 1.56 Å and 2.68 Å for Ni‐N and Ni‐Ni paths, respectively, suggesting the successful coordination between Ni with N (Figure 2h). The CN value of Ni–N (3.98) revealed the coordination‐saturated structure of Ni–N_4_. Moreover, the WT contour plot of Co_x_Ni_3‐x_(HITP)_2_/BNSs‐P shows the two maximum intensities at 4.02 and 5.73 Å^−1^ (Figure 2k,l, and Figure S22b, Supporting Information) for Ni–N and Ni–Ni paths, respectively. This observation further confirms that plasma treatment can generate unsaturated metal coordination, which is consistent with the EPR and Raman results.

The eNRR performance was evaluated in an H‐type cell with an N_2_‐saturated 0.1 m HCl solution. For monometallic M_3_(HITP)_2_ (M=Co, Ni, Cu, Mn, Fe), Co_3_(HITP)_2_ had the highest NH_3_ yield of 21.08 µg h^−1^ mg_cat._^−1^ and a FE of 18.8% (Figure S23, Supporting Information). Bimetallic Co_x_M_3‐x_(HITP)_2_ (M = Ni, Cu, Mn, Fe) and Co_x_Ni_3‐x_(HITP)_2_ shows the highest NH_3_ yield of 33.27 µg h^−1^ mg_cat._^−1^ and a FE of 22.8% (Figure S24, Supporting Information). The superior eNRR performance of Co_x_Ni_3‐x_(HITP)_2_ compared to Co_3_(HITP)_2_ and Ni_3_(HITP)_2_ is ascribed to the variation of electron density of Co sites caused by the introduction of Ni. To gain deeper insight into the catalytic mechanism of Co_x_M_3‐x_(HITP)_2_, density functional theory (DFT) calculations were performed to probe their electronic properties (Figure S25, Supporting Information). To trigger the eNRR by the adsorption and activation of N_2_ molecules, Co_3_(HITP)_2_ can fill the conduction band of Co and transfer electrons to the π* orbital of N_2_ after obtaining electrons. In contrast, the *σ* orbital of N_2_ feeds back to the valence band of Co. To investigate the influence of the electronic properties of Co_x_M_3‐x_(HITP)_2_ on the activation of N_2_ molecules, the conduction band minimum (CBM) and valence band maximum (VBM) were calculated. The CBMs of Co_x_M_3‐x_(HITP)_2_ (M=Ni, Cu, Mn, Fe) are mainly distributed on the Co atoms (Figure S25a–e, Supporting Information), indicating that the electrons can be transferred to the π* orbital of N_2_ via CBM. The VBMs of Co_x_Mn_3‐x_(HITP)_2_ (Figure S24d, Supporting Information) and Co_x_Ni_3‐x_(HITP)_2_ (Figure S25e, Supporting Information) are not distributed on the Co atoms, which is favorable for receiving N_2_ *σ* electrons. The VBM distribution around the Co atoms in Co_x_Ni_3‐x_(HITP)_2_ is less than that in Co_x_Mn_3‐x_(HITP)_2_, which is more conducive to accepting electrons. This effect can activate N_2_ molecules and enhance the eNRR activity, which is consistent with the electrochemical experiments.

**Figure 3:**
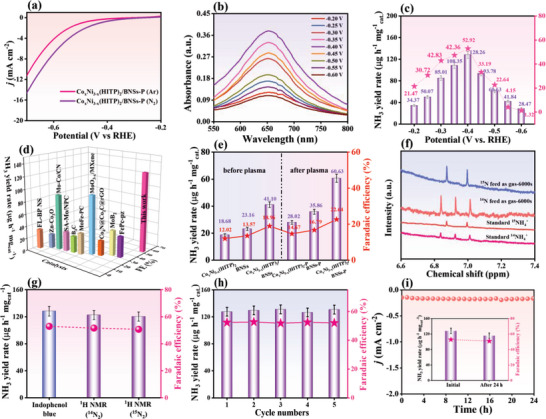
a) LSV curves of Co_x_Ni_3‐x_(HITP)_2_/BNSs‐P in N_2_‐ and Ar‐saturated solutions. b) UV‐vis absorption spectra of Co_x_Ni_3‐x_(HITP)_2_/BNSs‐P in 0.1 M N_2_‐saturated HCl solution at various applied potentials. c) Dependence of NH_3_ yield and FE of Co_x_Ni_3‐x_(HITP)_2_/BNSs‐P at each applied potential in N_2_‐saturated 0.1 m HCl with the NRR measurement time of 6000 s. d) Comparison of NH_3_ yield rate of the eNRR catalysts in 0.1 m HCl, *n* = 3. e) The eNRR performances of different catalysts at −0.5 V versus RHE. f) ^15^N_2_, ^14^N_2_ isotope labeling and indophenol blue experiments. g) Comparison of NH_3_ yields calculated by NMR and indophenol blue tests. All current responses are presented as mean values ±SD, *n*  =  3. h) Cycling stability of Co_x_Ni_3‐x_(HITP)_2_/BNSs‐P at −0.4 V versus RHE. i) Chronoamperometry tests for 24 h (inset: NH_3_ yield and FE of original and post‐eNRR electrolysis).

The linear sweep voltammetry curves for Co_x_Ni_3‐x_(HITP)_2_/BNSs‐P measured in 0.1 M N_2_‐saturated HCl solution from 0 to −0.80 V versus RHE exhibit a significantly larger current density than that in 0.1 M Ar‐saturated HCl solution (**Figure** **3**a), illustrating the facilitated electrocatalytic ability toward the eNRR of Co_x_Ni_3‐x_(HITP)_2_/BNSs‐P. The concentrations of the NH_4_^+^ and N_2_H_4_ products are quantified by UV‐vis spectroscopy using the indophenol blue method (Figures S26 and S27, Supporting Information). To eliminate the interference of the background signal, the corresponding NH_3_ concentration in the electrolyte was determined after continuously bubbling Ar or N_2_ during electrolysis for 6000 s. Control experiments were performed under an open circuit using bare carbon paper in an N_2_‐saturated 0.1 m HCl solution under the same conditions. Figure S28 (Supporting Information) shows no NH_3_ formation, suggesting that the produced NH_3_ originated entirely from N_2_ gas. To quantitatively analyze the eNRR performance of Co_x_Ni_3‐x_(HITP)_2_/BNSs‐P, time‐dependent current density curves were recorded at various potentials. The curves remain stable within 6000 s (Figure S29, Supporting Information). The UV‐vis absorption curves of the electrolytes tested at different potentials are shown in Figure 3b, while the corresponding NH_3_ yield and FE are illustrated in Figure 3c. Co_x_Ni_3‐x_(HITP)_2_/BNSs‐P shows a large NH_3_ yield of 60.63 µg h^−1^ mg_cat._^−1^ in 0.1 m HCl at −0.50 V (vs RHE) with a high FE of 22.64%. These values are remarkably higher than those of Co_x_Ni_3‐x_(HITP)_2_/BNSs (NH_3_ yield of 41.1 µg h^−1^ mg_cat._^−1^ and FE of 18.96%). However, when the negative shift of the voltage exceeds −0.50 V, both NH_3_ production and FE significantly decrease because of the competitive HER process.

As mentioned above, the enhancement of defects and vacancies of Co_x_Ni_3‐x_(HITP)_2_/BNSs‐P can effectively enhance the NRR kinetics to inhibit the HER, thus increasing the FE of the eNRR. The highly active species generated by plasma treatment (such as reactive oxygen^[^ ^30^ ^]^ and defects^[^ ^31^ ^]^) can disassemble the partial, weak metal‐N bonds on the external framework of the MOF, thus increasing the defect density. Moreover, Figure S30i–vi (Supporting Information) shows that the water contact angle of the Co_x_Ni_3‐x_(HITP)_2_/BNSs‐P heterojunction is ≈123.7°, which is markedly greater than that of Co_x_Ni_3‐x_(HITP)_2_/BNSs (88.3°). In this regard, plasma modification not only created defects but also improved the hydrophobicity of the catalyst surface, which suppressed the HER. Remarkably, Co_x_Ni_3‐x_(HITP)_2_/BNSs‐P shows higher NH_3_ yield along with FE than the known MOF‐based eNRR catalysts (Figure 3d and Table S3, Supporting Information), such as MoFe‐PC (34.23 µg h^−1^ mg_cat._^−1^ and 16.83%),^[^ ^32^ ^]^ FePc‐pz (33.6 µg h^−1^ mg_cat._^−1^, 31.9%),^[^ ^33^ ^]^ and SA‐Mo/NPC (31.5 µg h^−1^ mg_cat._^−1^, 14.6%)^[^ ^34^ ^]^ as well as other types of state‐of‐the‐art eNRR catalysts including MoO_3‐x_/MXene (31.5 µg h^−1^ mg_cat._^−1^, 14.6%),^[^ ^35^ ^]^ MoB_2_ (31.5 µg h^−1^ mg_cat._^−1^, 14.6%),^[^ ^36^ ^]^ and Mo‐Co/NC (89.8 µg h^−1^ mg_cat._^−1^, 13.5%).^[^ ^37^ ^]^

No trace of N_2_H_4_ product suggests that the eNRR for Co_x_Ni_3‐x_(HITP)_2_/BNSs‐P is highly selective (Figure S31, Supporting Information). Further, the NH_3_ production (41.0 µg h^−1^ mg_cat._^−1^) and FE (18.96%) of Co_x_Ni_3‐x_(HITP)_2_/BNSs for the eNRR are higher than those of the individual Co_x_Ni_3‐x_(HITP)_2_ (18.68 µg h^−1^ mg_cat._^−1^ and 12.02%) and BNSs (23.16 µg h^−1^ mg_cat._^−1^ and 13.57%) (Figure S3e and S32, Supporting Information). In addition, Co_x_Ni_3‐x_(HITP)_2_/BNSs shows the superior eNRR performance compared to that of BNSs‐Co_x_Ni_3‐x_(HITP)_2_ (19.74 µg h^−1^ mg_cat._^−1^ and 14.54%) and Co_x_Ni_3‐x_(HITP)_2_‐BNSs (26.53 µg h^−1^ mg_cat._^−1^ and 16.71%) (Figure S33, Supporting Information). This reveals that an effective junction can be formed at the interface between Co_x_Ni_3‐x_(HITP)_2_ and BNSs, in which the electron density of the active metal sites of Co_x_Ni_3‐x_(HITP)_2_ can be modulated by BNSs. In addition, an interface engineering strategy can regulate N_2_ adsorption and activation.^[^ ^11^ ^]^ All these factors may jointly contribute to improving the NH_3_ yield and FE, which will be discussed in the following DFT computations. Moreover, after plasma modification, both Co_x_Ni_3‐x_(HITP)_2_‐P and BNSs‐P exhibited improved NH_3_ yields and Faradaic efficiencies compared to the pristine samples.

An isotope labeling experiment was performed to verify whether the detected NH_3_ originated exclusively from the added N_2_ gas. The ^1^H NMR spectra of the eNRR experiment with ^15^N_2_ gas as the feeding gas and the commercial ^15/14^NH_4_^+^ sample (Figure 3f) shows a clear ^15^NH_4_^+^ signal when using ^15^N_2_ as the feeding gas, but no signal when using Ar. This observation confirms that NH_3_ is mainly produced from the eNRR. Moreover, the yields of ^14^N‐ labeled and ^15^N‐labeled NH_3_ quantified by ^1^H NMR (Figures S34 and S35, Supporting Information) suggest that the amount of NH_3_ produced by the Co_x_Ni_3‐x_(HITP)_2_/BNSs‐P catalyst is 120.31 ± 2.37 µg h^−1^ mg_cat._^−1^ in the 0.1 m HCl electrolyte, which is close to the result obtained by the indophenol blue method (128.26 ± 2.27 µg h^−1^ mg_cat._^−1^) (Figure 3g).

Co_x_Ni_3‐x_(HITP)_2_/BNSs‐P shows a comparable eNRR performance over five successive cycles (Figure 3h and Figure S36, Supporting Information). Additionally, the alternating eNRR cycling test was conducted at −0.4 V versus RHE in the N_2_ and Ar‐saturated electrolytes (Figure S37, Supporting Information). The NH_3_ yield rate of Co_x_Ni_3‐x_(HITP)_2_/BNSs‐P was significant and stable in each cycle in the N_2_‐saturated electrolyte, while no NH_3_ was observed in an Ar‐saturated electrolyte, confirming the eNRR process over Co_x_Ni_3‐x_(HITP)_2_/BNSs‐P. The chronoamperometry plot at −0.4 V versus RHE for Co_x_Ni_3‐x_(HITP)_2_/BNSs‐P shows little change (Figure 3i) after continuous measurement for 24 h, affording the comparable NH_3_ yield and FE with the initial catalyst (Figure 3i, inset). This confirms the superior long‐term durability of the Co_x_Ni_3‐x_(HITP)_2_/BNSs‐P catalyst. Additionally, the water contact angle of Co_x_Ni_3‐x_(HITP)_2_/BNSs‐P before and after chronoamperometry tests were measured (Figure S38, Supporting Information). As expected, the contact angle of Co_x_Ni_3‐x_(HITP)_2_/BNSs‐P decreases to 103.1°, which also contributed to the performance degradation. Moreover, no substantial change is observed in the SEM and TEM images, PXRD pattern, and XPS spectra of the Co_x_Ni_3‐x_(HITP)_2_/BNSs‐P heterojunction (Figure S39, Supporting Information). Time‐dependent Inductively coupled plasma‐mass spectrometry was performed to determine the exact percentages of Co and Ni in 0.1 m HCl in the chronoamperometry tests. As indicated in Table S4, Supporting Information, the metal content in 0.1 m HCl increased with reaction time and approached stability. It indicates that the concentration of Co in 0.1 m HCl was slightly higher than that of Ni for the same duration, indicating that Co‐based species were the main active sites in the eNRR.

The charge‐transfer resistance (*R*_ct_) of Co_x_Ni_3‐x_(HITP)_2_/BNSs‐P was 29 Ω, clearly lower than that of Co_x_Ni_3‐x_(HITP)_2_/BNSs (40 Ω) and the individual components before and after plasma modification (Figure S40h and Table S5, Supporting Information). Cyclic voltammogram curves (Figure S40g, Supporting Information) were obtained over a small potential window of 0.2 to 0.3 V vs Ag/AgCl to evaluate the double‐layer capacitance (*C*_dl_). The capacitive currents, Δ*j*/2 (Δ*j* = *j*_a_ − *j*_c_, where *j*_a_ and *j*_c_ are the anodic and cathodic current densities at the same voltage, respectively) at 0.15 V versus Ag/AgCl, were plotted versus the scan rates. The *C*_dl_ value was calculated to be 6.96 mF cm^−2^ for Co_x_Ni_3‐x_(HITP)_2_/BNSs‐P, which is slightly larger than that of Co_x_Ni_3‐x_(HITP)_2_/BNSs (6.60 mF cm^−2^). This suggests that the enhanced oxygen vacancies and defects in the Co_x_Ni_3‐x_(HITP)_2_/BNSs‐P junction generated by plasma irradiation can promote electron transfer, improving eNRR performance.

**Figure 4:**
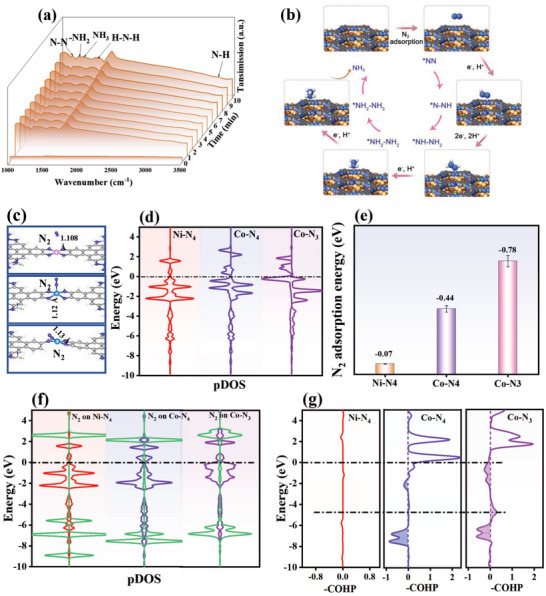
a) Electrochemical in situ FT‐IR spectra on Co_x_Ni_3‐x_(HITP)_2_/BNSs‐P during NRR. b) Mechanism of eNRR on Co_x_Ni_3‐x_(HITP)_2_/BNSs‐P. c) Side view of the structure for *N_2_ on Ni‐N_4_, Co‐N_4_, and Co‐N_3_. (gray: C, blue: N, light blue: Co, pink: Ni). d) N_2_ adsorption energy of Ni‐N_4_, Co‐N_4_, and Co‐N_3_. e) Partial density of states (pDOS) of Ni‐N_4_, Co‐N_4,_ and Co‐N_3_. f) pDOS of N_2_ adsorption on Ni‐N_4_, Co‐N_4_, and Co‐N_3_. g) Projected crystal orbital Hamilton populations (pCOHP) for N–N bonded to Ni‐N_4_, Co‐N_4_, and Co‐N_3_.

To clarify the details of the conversion of N_2_ to NH_3_ on Co_x_Ni_3‐x_(HITP)_2_/BNSs‐P, time‐dependent in situ FTIR spectra were determined at −0.4 V versus RHE for 6000 s in 0.1 M N_2_ saturated HCl electrolyte. The absorption peak at 1171 cm^−1^ (**Figure** **4**a) is ascribed to the N–N stretching bond, indicating breakage of the N≡N bond on the working electrode surface.^[^ ^38^ ^]^ The adsorption peaks at 1272, 1442, and 3278 cm^−1^ are ascribed to ‐NH_2_ vibration, H–N–H bending, and N–H stretching vibrations, respectively.^[^ ^39^ ^]^ Notably, the intensity of these peaks increased with the reaction time. Clearly, N_2_H*_y_* (1≦*y*≦4) species were generated on the surface of Co*_x_* Ni_3‐x_(HITP)_2_/BNSs‐P during the eNRR process.^[^ ^38^ ^]^ Most importantly, an absorption peak at 1637 cm^−1^ is observed for N_2_ molecules adsorbed on the catalyst surface.^[^ ^40^ ^]^ According to the in situ FTIR spectra of the intermediate products, it can be concluded that the eNRR on Co_x_Ni_3‐x_(HITP)_2_/BNSs‐P can follow alternate pathways (Figure 4b).

Additional DFT calculations were performed on Co_x_Ni_3‐x_(HITP)_2_/BNSs‐P and the Co‐N_4_, Co‐N_3_, and Ni‐N_4_ models (Figure S41, Supporting Information). The N_2_ adsorption behaviors of different models are also explored (Figure 4c), as sufficient N_2_ adsorption and activation are necessary prerequisites for an efficient eNRR.^[^ ^35^ ^]^ The N_2_ molecules prefer to take the side‐on configuration on Co‐N_3_, which has an increased N–N bond length (1.13 Å) compared with Co‐N_4_ and Ni‐N_4_. The defects in Co_x_Ni_3‐x_(HITP)_2_/BNSs caused by plasma treatment can promote N_2_ adsorption. Clearly, the junction of BNSs with Co_x_Ni_3‐x_(HITP)_2_ can effectively increase the number of Co‐N_3_ active sites to enhance eNRR activity. To achieve more insight into the N_2_ activation ability of Co_x_Ni_3‐x_(HITP)_2_/BNSs and Co_x_Ni_3‐x_(HITP)_2_/BNSs‐P, the projected density of states (pDOS) and projected crystal orbital Hamilton population (pCOHP) of Co‐N_4_, Ni‐N_4_, and Co‐N_3_ before and after the N_2_ adsorption were calculated. The *d* state of Co‐N_3_ (Figure 4d) shifts toward the Fermi level compared to those of Co‐N_4_ and Ni‐N_4_ before N_2_ adsorption. This suggests that the defects caused by the plasma treatment can modulate the electronic structure of Co, thereby improving the conductivity and accelerating the electron transfer of the catalyst, as confirmed by the smaller *R*_ct_ (29 Ω) of Co_x_Ni_3‐x_(HITP)_2_/BNSs‐P (Table S4, Supporting Information). After N_2_ adsorption (Figure 4f), more pDOS of Co‐*d* for Co‐N_3_ involves an overlapping region with N‐2*p* near the Fermi level. The Ni‐*d* of Ni‐N_4_ hardly overlaps with the N_2_‐*p* orbital, indicating that the adsorption of N_2_ by Ni‐N_4_ is weak. The adsorption energies of N_2_ molecules on Ni‐N_4_, Co‐N_4_, and Co‐N_3_ were −0.07, −0.41, and −0.78 eV, respectively (Figure 4e). This result further indicates that Co_x_Ni_3‐x_(HITP)_2_/BNSs‐P adsorbs N_2_ more strongly than Co_x_Ni_3‐x_(HITP)_2_/BNSs. From ‐COHP (Figure 4g), the bonding orbital of Co‐N_3_ (−4 eV) is significantly enhanced compared with Co‐N_4_ because of the formation of a bonding orbital between N_2_ and Co‐N_3_. This leads to charge transfer from the *π* bond of N≡Ν to the Co–N bond, weakening the N≡Ν bond and reducing N_2_ adsorption. Therefore, the antibonding orbitals of the N_2_ molecules can be filled after N_2_ adsorption. The number of electrons in the antibonding orbitals of N–N on Co‐N_3_ is greater than that on the Co‐N_4_ site, which promotes the efficient adsorption of N_2_ on Co‐N_3_. In addition, as B is widely used as a highly efficient active site for the eNRR,^[^ ^41^ ^]^ Co_x_Ni_3‐x_(HITP)_2_/BNSs‐P can be used for N_2_ adsorption by introducing a large number of O vacancies on BNSs after plasma treatment. Thus, Co_x_Ni_3‐x_(HITP)_2_/BNSs‐P comprises multiple active sites of Co‐N_3_, O vacancies, and B atoms that drive the eNRR.

Based on the above results, the superior eNRR performance of Co_x_Ni_3‐x_(HITP)_2_/BNSs‐P can be attributed to the following points: i) the high porosity and nanosheet structure of Co_x_Ni_3‐x_(HITP)_2_/BNSs‐P favor high adsorption of N_2_, which also stabilizes the intermediate products during the eNRR procedure;^[^ ^42^ ^]^ in contrast, the enhanced Lewis acidity caused by oxygen vacancies, defects, and B sites of oxidized BNSs can further strengthen the activation of N_2_ at the junction interface;^[^ ^18^ ^]^ ii) the superior conductivity of both Co_x_Ni_3‐x_(HITP)_2_ and BNSs facilitates the electron transfer, and the hydrophobicity of the heterojunction efficiently suppresses the HER, thus improving the FE;^[^ ^43^ ^]^ iii) multiple active sites in the Co_x_Ni_3‐x_(HITP)_2_/BNSs‐P heterojunction, such as unsaturated Co‐N_3_, O vacancies, and B‐B bonds afford fast kinetics and a high catalytic ability for the eNRR.

Given the superior NH_3_ production and FE, a rechargeable Zn‐N_2_ battery was assembled using Co_x_Ni_3‐x_(HITP)_2_/BNSs‐P as the cathode and Zn foil as the anode for self‐powered N_2_ reduction and energy transfer. N_2_ and Ar gases were separately bubbled into the cathode cell, which was separated from the anode chamber using a Nafion 117 membrane (Figure S42, Supporting Information). During the discharge process of this rechargeable Zn‐N_2_ battery, the reactions on the electrodes included the following:

(1) $$\mathrm{The}\;\mathrm{cathode}\;\mathrm{reaction}:N_{2}+6H^{+}+6e^{-}\to 2NH_{3}$$

(2) $$\mathrm{The}\;\mathrm{anode}\;\mathrm{reaction}:\mathrm{Zn}\to {\mathrm{Zn}}^{2+}+2e^{-}$$

(3) $$\mathrm{The}\;\mathrm{overall}\;\mathrm{reaction}:3\;\mathrm{Zn}+N_{2}+6H^{+}\to 3Zn^{2+}+2NH_{3}$$

For battery charging, the half‐reactions at the electrodes can be expressed as follows:

(4) $$\mathrm{The}\;\mathrm{anode}\;\mathrm{reaction}:2NH_{3}+6Cl^{-}\to 6\mathrm{HCl}+N_{2}+6e^{-}$$

(5) $$\mathrm{The}\;\mathrm{cathode}\;\mathrm{reaction}:{\mathrm{Zn}}^{2+}+2e^{-}\to \mathrm{Zn}$$

(6) $$\begin{array}{c} \mathrm{Overall}\;\mathrm{reaction}:3Zn^{2+}+2NH_{3}+6Cl^{-}\to 3\;\mathrm{Zn}+N_{2}+6\mathrm{HCl} \end{array}$$

**Figure 5:**
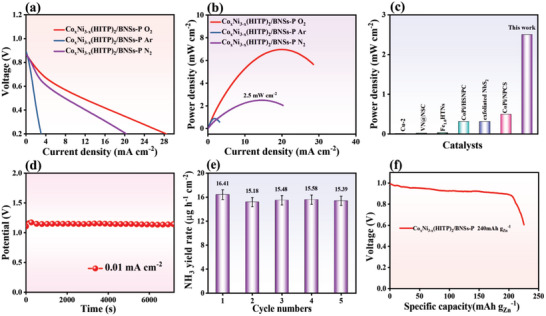
a) Discharging polarization curves of Zn‐N_2_ battery with Co_x_Ni_3‐x_(HITP)_2_/BNSs‐P. b) Power density plot of Co_x_Ni_3‐x_(HITP)_2_/BNSs‐P. c) Comparison of NH_3_ yields of different catalysts. d) The discharge curve of Zn‐N_2_ battery with Co_x_Ni_3‐x_(HITP)_2_/BNSs‐P at 0.01 mA cm^−2^ for 20 h. e) The eNRR performance of Zn‐N_2_ battery with Co_x_Ni_3‐x_(HITP)_2_/BNSs‐P for five repeated tests. f) Discharge curve at a current density of 0.01 mA cm^−2^.

The galvanostatic discharge polarization plot for the cathode fed with N_2_ showed a distinct discharge current density at approximately 1.75 V with a maximum value of 9.5 mA cm^−2^ at 0 V versus Zn^2+^/Zn (**Figure** **5**a). The high discharge current density of the Zn‐N_2_ battery showed a clear eNRR when feeding N_2_ to the catholyte during discharge. Figure 5b shows that the Co_x_Ni_3‐x_(HITP)_2_/BNSs‐P‐assembled Zn‐N_2_ battery possessed the highest power density of 5.4 mW cm^−2^, which is remarkably larger than that of the battery fed by Ar. This Zn‐N_2_ battery shows significantly higher power density than earlier reported examples (Figure 5c and Table S5, Supporting Information), such as CoPi/NPCS (0.49 mW cm^−2^),^[^ ^44^ ^]^ exfoliated NbS_2_ nanosheets (0.31 mW cm^−2^),^[^ ^45^ ^]^ and VN nanodots dispersed in graphitized carbon (16.42 µW cm^−2^).^[^ ^46^ ^]^ To further examine the stability of this Zn‐N_2_ battery, the discharge curve was taken at 0.01 mA cm^−2^ for 7000 s (Figure 5d). No substantial change in the discharge voltage occurred, suggesting excellent stability. After being discharged for 20 h, the electrolyte was analyzed using the indophenol blue method to evaluate the yield rate of NH_3_. Notably, the Zn‐N_2_ battery (Figure 5e) shows excellent reproducibility with an almost constant NH_3_ yield for each of the five cycles at 1.16 V versus Zn^2+^/ Zn. During each discharge, a high NH_3_ yield of 15.48 µg h^−1^ mg_cat._^−1^ was obtained, which reveals the excellent cycling stability of NH_3_ production during continuous operation of the battery system. Additionally, a high energy density of 240 mA h g^−1^ was achieved at 0.01 mA cm^−2^ (Figure 5f).

## 3 Conclusion

A new type of *p‐n* heterojunction with dual Co‐N_3_ and B‐B active sites was constructed based on a semiconductive MOF and BNSs by in situ solution plasma treatment. The numerous defects, oxygen vacancies, unsaturated metal‐N sites, and functional B‐related bonds in Co*_x_* Ni_3‐_ *_x_* (HITP)_2_/BNSs‐P contributed to its outstanding NH_3_ yield of 128.26 ± 2.27 µg h^−1^ mg_cat._^−1^ and FE of 52.92 ± 1.83% in 0.1 m HCl solution. Co*_x_* Ni_3‐_ *_x_* (HITP)_2_/BNSs‐P can also serve as the cathode of a Zn‐N_2_ battery, achieving an unprecedented density of 5.4 mW cm^−2^. The in situ FT‐IR characterization of the intermediate products confirmed that the eNRR arose from the Co_x_Ni_3‐x_(HITP)_2_/BNSs‐P heterojunction. Moreover, DFT calculations showed that the adsorption and activation of N_2_ molecules on the heterojunction were significantly enhanced by the plasma treatment, thus improving the eNRR performance. The results reveal that constructing dual‐active heterojunctions by interface and defect engineering is a promising, clean approach to achieving high NH_3_ yield and FE for the eNRR.

## Conflict of Interest

The authors declare no conflict of interest.

## Supporting information

Supporting Information

Supplemental Table 1

## Data Availability

The data that support the findings of this study are available in the supplementary material of this article.
