# Supplementary material for: Plasma‐Assisted Defect Engineering on p‐n Heterojunction for High‐Efficiency Electrochemical Ammonia Synthesis
Source: Adv Sci (Weinh). 2023 Jan 22;10(8):2205786. doi: 10.1002/advs.202205786 (PMC10015844; doi:10.1002/advs.202205786)
Supplement: Supplementary file 1 — Supporting Information [file ADVS-10-2205786-s002.pdf]

ADVANCED SCIENCE
Open Access

Supporting Information

for *Adv. Sci.*, DOI 10.1002/advs.202205786

Plasma-Assisted Defect Engineering on *p-n* Heterojunction for High-Efficiency Electrochemical Ammonia Synthesis

Jiameng Liu, Linghao He, Shuangrun Zhao, Sizhuan Li, Lijun Hu, Jia-Yue Tian, Junwei Ding, Zhihong Zhang and Miao Du**

Supporting Information

Plasma-Assisted Defect Engineering on *p-n* Heterojunction for High-Efficiency Electro-chemical Ammonia Synthesis

Jiameng Liu, Linghao He, Shuangrun Zhao, Sizhuan Li, Lijun Hu, Jia-Yue Tian, Junwei Ding, Zhihong Zhang, and Miao Du**

Experimental section

1. Materials and methods

$CoCl_2·6H_2O$ and $NiCl_2·6H_2O$ were obtained from Sinopharm Chemical reagent Co., Ltd. The ligand of 2,3,6,7,10,11-hexaaminotriphenylene (HATP) was purchased from Solarbio Shanghai Yushu Chemical Technology Co., Ltd. Paradimethylaminobenzaldehyde ($C_9H_{11}N_O$, 99%) was obtained from Shanghai Yuanye Bio-Technology Co., Ltd. Sodium borohydride ($NaBH_4$, 98%) and sodium citrate ($Na_3C_6H_5O_7·2H_2O$, 99.5%) were from Aladdin. Hydroquinone (99%) and NaOH (96%) were purchased from Tianjin Kermel Chemical Reagent Co., Ltd. Hydrochloric acid (HCl, 36~38%) and acetone (99.5%) were obtained from Luoyang Haohua Chemical Reagent Co., Ltd. Ethanol (99%), sodium sulfate (99%), and ammonium chloride (NH_4Cl, 99%) were obtained from Tianjin Damao Chemical Trading Co., Ltd. Sodium nitroprusside ($C_5H_4FeN_6Na_2O_3$, 99%) and salicylic acid ($C_7H_6O_3$, 99.5%) were purchased from Ron Reagent, while hydrazine hydrate (N_2H_4, 5% HCl) was obtained from Guobiao (Beijing) Testing and Certification, Co., Ltd. Ammonia (NH_4OH, 25%) was from Fortune Chemical Reagent Co., Ltd, and NaClO (6~14% active chlorine basis) was obtained from Maclin. Further, $^{14}N_2$ gas (99.99%) and Ar gas (99.99%) were obtained from Huanyu Co., Ltd, while $^{15}N_2$ gas (99.99%) was obtained from Tianjin Taiya Co., Ltd. All aqueous solutions were prepared with deionized water, and all chemicals were of analytical grade and used without further purification.

2. Preparation of MOFs

The synthesis of monometallic MOFs $M_3(HITP)_2$ (M = Co, Ni, Cu, Mn, Fe) and bimetallic MOF $Co_xNi_{3-x}(HITP)_2$ was referred to our previous work,[1] but with a slight change. Briefly, HATP (10 mg) was dissolved in water (5 mL) to form Solution A, while $NiCl_2·6H_2O$ (6.6 mg) and $CoCl_2·6H_2O$ (6.6 mg) were dissolved in water (10 mL) to form Solution B. Subsequently, solutions A and B were mixed, followed by slowly adding ammonia (14 M, 0.6 mL). After fully mixed, the resulting solution was transferred into a round neck flask (100 mL) and then stirred for 1 day at 120 °C. The product was separately washed three times with acetone and water, and dried in a vacuum oven at 60 °C. Likely, $Co_xCu_{3-x}(HITP)_2$, $Co_xMn_{3-x}(HITP)_2$, and $Co_xFe_{3-x}(HITP)_2$, were prepared using the same method, only by replacing $NiCl_2·6H_2O$ with $CuCl_2·6H_2O$, $MnCl_2·6H_2O$, and $FeCl_2·6H_2O$, respectively.

3. Preparation of boron nanosheets (BNSs)

BNSs were obtained by calcining sodium borohydride powder at high temperature. First, 200 mg of sodium borohydride powder was heated from room temperature to 490 °C at a rate of 10 °C min^{-1} for 2 h, and continuously heated to 550 °C at a rate of 5 °C min^{-1} for 30 min to form stable intermediates. Then, the intermediate was heated to 600 °C at a rate of 5 °C min^{-1} for 30 min to obtain BNSs. Notably, the whole process was taken under the protection of Ar. After cooling down, the product was washed with water to remove unreacted precursors and dried at 60 °C under vacuum.

4. Plasma-modification procedure

$Co_xNi_{3-x}(HITP)_2$/BNSs-P was obtained according to the following procedure. Typically, 2 mg $Co_xNi_{3-x}(HITP)_2$ was dispersed in 1.9 mL water in a glass vial, following by sonicated for 10 min. Then, 3 mg BNSs were added into the $Co_xNi_{3-x}(HITP)_2$ suspension and sonicated for 30 min (denoted as $Co_xNi_{3-x}(HITP)_2$/BNSs). Subsequently, the glass vial was placed into the plasma chamber and irradiated with a continuous wave for 5 min at a plasma input power of 200 W under a pressure of 0.1 Pa. Afterward, 100 μL of 5 wt% Nafion was added to the obtained solution and sonicated for 30 min to form a homogeneous ink. The prepared $Co_xNi_{3-x}(HITP)_2$/BNSs-P catalyst was stored for further electrochemical measurements. In addition, the $Co_xNi_{3-x}(HITP)_2$ suspension (40 μL, 2 mg mL^{-1}) was coated on the carbon paper, and dried in air. After that, the BNSs suspension (40 μL, 2 mg mL^{-1}) was dropped on the $Co_xNi_{3-x}(HITP)_2$-modified carbon paper (represented by $Co_xNi_{3-x}(HITP)_2$-BNSs). In addition, the modification step of the carbon paper was upside down. The achieved electrode was then denoted as BNSs-$Co_xNi_{3-x}(HITP)_2$.

5. Electrocatalytic nitrogen reduction reaction (eNRR) measurements

Electrochemical measurements were performed on an H-type-cell equipped with a CHI 760E workstation (CH Instruments, Inc., Shanghai, China), which was separated by the Nafion 117 membrane. The $Co_xNi_{3-x}(HITP)_2$/BNSs-P electrode, Ag/AgCl (Saturated KCl) electrode and graphite rod work as the working, reference, and counter electrodes, respectively, using 0.1 M HCl as the electrolyte. The prepared catalyst (2 mg) was dispersed in 0.95 mL of water, followed by ultrasonication for 30 min to form homogeneous dispersion. Then, the suspension was mixed with 0.05 mL of 5% Nafion solution. Subsequently, 80 μL of the mixture was dipped onto carbon paper (1 × 1 cm^2) with a loading mass of 0.16 mg cm^{-2}, and dried at room temperature. Before each eNRR process, the used electrolyte was pretreated by a refrigerated pumping process for three cycles to ensure removal of air. After that, the self-built H-cell was

bubbled continuously with N_2 gas for at least 30 min. Subsequently, the gas was maintained throughout electrochemical reaction. Cyclic voltammetry (CV) tests were carried out at a scan rate of 50 mV s^{-1} at the applied potential of -0.6 V and -0.2 V vs. RHE in N_2-saturated electrolyte. Potentiostatic measurements were taken at a series of applied potentials including -0.2, -0.3, -0.4, -0.5, and -0.6 V vs. RHE for 6000 s under constant room temperature. The potentials were converted to RHE scale via calibration with the following equation: E (vs. RHE) = E (vs. Ag / AgCl) + 0.22 V.

6. Statistical Analysis

All measurements were made in triplicate and presented as mean±standard deviation. Statistical significance between two groups was analyzed by the Student's t-test. In addition, there was no significant difference between the two electrodes in parallel test for 3 times ($P=0.909>0.005$), indicating the reliability of the data.

7. Detection of ammonia

Indophenol blue method was used to estimate the concentration of ammonia in the electrolyte containing 0.1 M HCl after the electrochemical measurement for 6000 s. The color reagent system was prepared: solution A, 1 M NaOH solution containing 5 wt% salicylic acid and 5 wt% sodium citrates; solution B, 0.05 M NaClO; solution C, 1 wt% $C_5FeN_6Na_2O$ (sodium nitroferricyanide) aqueous solution. The concentration-absorbance curves were established using a series of standard ammonia solutions. First, 2 mL of post-tested electrolyte solution was removed from electrochemical system, followed by the sequential addition of 1 mL of solution A, and 0.2 mL of solution C. After keeping for 2 h at room temperature, the absorption spectrum was measured on an ultraviolet-visible spectrophotometer. The absorbance intensity at 655 nm was utilized to estimate the yield of ammonia based on the standard curve.

8. Detection of hydrazine

Concentration of hydrazine was spectrophotometrically determined using Watt and Chrisp method. A mixture of para-(dimethylamino) benzaldehyde (5.99 g), HCl (concentrated, 30 mL), and ethanol (300 mL) was used as a color reagent. 3 mL of solution was taken out from the electrochemical reaction vessel and the above color reagent was added. The mixture was kept stirring for 10 min at room temperature. The amount of hydrazine formed during the electrolysis was determined based on the absorbance intensity at 455 nm. The concentration-absorbance curve was established by standard hydrazine solution, which contains the same concentration of HCl as used in each electrolysis experiment.

9. Isotope labeling experiment

Before the test, the labeled $^{15}N_2$ (99 % ^{15}N) as the feed gas was pre-purified through flowing into NaOH solution, $KMnO_4$ solution, and then passing through a volumetric flask with two-thirds of Na_2SO_4 solution to remove any N contamination, and then the gas flowed out was collected. In the eNRR measurement, Ar gas was purged to the cathodic cell to remove impurity gas and purge for 30 min with the gas to be tested. After testing in 0.1 M HCl electrolyte for 6000 s, 20 mL of the electrolyte was taken out and then concentrated to 2 mL by heating via reduced pressure distillation. Afterward, 0.9 mL of the resulting solution was mixed with 0.10 mL DMSO-d^6 for the ^1H NMR measurements.

10. Calculation of ammonia yield rate and Faradaic efficiency

The Faradaic efficiency (FE) for the eNRR was defined as the amount of electric charge used for NH_3 synthesis divided by the total charge passed through the electrodes during electrolysis.

$$Y = 0.3011X + 0.0322$$

$$r(NH_3) = 3600 \times \frac{X \times 30}{6000}$$

Among them, Y is the value obtained by UV spectra testing the solution after color development, and r(NH_3) is the rate of NH_3 production.

Assuming three electrons were required to produce one NH_3 molecule, the FE was calculated as:

$$FE(NH_3) = \frac{3 \times \mathrm{F} \times \frac{[NH_3]}{17} \times V}{1000000 \times \int idt}$$

where [NH_3] is the measured concentration of NH_4^+, V is the volume of HCl electrolyte in the cathode chamber, i is the instantaneous current measured by chronoamperometry.

11. Characterizations

Field emission scanning electron microscopy (FE-SEM) images were obtained with a JSM-6700F (JEOL) operating at 5 kV in LABE mode. TEM images were obtained with a Tecnai F20 electron microscope operated at 80 kV. X-ray photoelectron spectroscopy (XPS) was taken to analyze the chemical compositions and bond characters by using PHI Quantera SXM with monochromatic Mg X-ray radiation source. PXRD patterns were recorded on a Bruker GADDS XRD diffractometer with Cu KĮ radiation to obtain the structures of composites. ^1H

NMR spectra were recorded on a Bruker NMR spectrometer (400 MHz) with chemical shifts reported as ppm in DMSO-d6. The work functions can be estimated as $\Phi = h\nu - (E_{cutoff} - E_{onset})$, where hν is the photon energy of the excitation light (21.2 eV). Electron paramagnetic resonance (EPR) measurements were conducted on a Bruker ESP-300 spectrometer. Thermogravimetric analyses (TGA) were measured on a Perkin-Elmer STA 6000 thermogravimetric analyzer under N_2 conditions from room temperature to 800 °C with a heating rate of 10 °C min^{-1}. Nitrogen absorption-desorption tests were performed on a Micromeritics ASAP 2020 adsorption apparatus at 77 K up to 1 bar. The porosity including specific Langmuir and Brunauer-Emmett-Teller (BET) surface areas, pore volume, and pore size was obtained by analyzing nitrogen adsorption-desorption isotherms with Micromeritics ASAP 2020 built-in software. ^1H NMR spectra were recorded on a Bruker NMR spectrometer (400 MHz) with chemical shifts reported as ppm in DMSO-d^6. Metal atomic content in samples was conducted using a Thermo Scientific iCAP 6500 model inductively coupled plasma-mass spectrometry. Synchrotron X-ray absorption spectroscopy (XAS) data were processed and analyzed using the Demeter software package. A linear function was subtracted from the pre-edge region, then the edge jump was normalized using Athena software. The χ(k) data were isolated by subtracting a smooth, polynomial approximating the absorption background of an isolated atom. The k weighted χ(k) data were Fourier transformed after applying window function. The global amplitudes were obtained by nonlinear fitting, with least-squares refinement, of the EXAFS equation to the Fourier-transformed data in R-space, using Artemis software.

12. Computational details

Density functional theory (DFT) calculation was performed by using the $Dmol^3$ code.[2] The exchange and correlation energies were determined with the Perdew-Burke-Ernzerhof (PBE) functional.[3] The DFT semi-core pseudo potentials (DSPPs) core treatment is implemented for relativistic effects, which replaces core electrons by a single effective potential and introduce some degree of relativistic correction into the core. Moreover, the double numerical plus polarization (DNP) is chosen as the basis set.[4] Among the basis sets provided by $dmol^3$ code, the DNP basis set has the best accuracy and highest computational cost. A thermal smearing of 0.005 Ha (1 Ha = 27.21 eV) to the orbital occupation is applied to speed up electronic convergence. The convergence tolerance of electronic structure and geometry optimization are 1×10^{-6} Ha, 2×10^{-3} Ha/Å, respectively. The global orbital cutoff is 4.5 Å. In the CBM and VBM orbit calculations, the grid interval and boundary were set to 0.1 Å and 5.0 Å, respectively. The crystal orbital Hamilton population (COHP) analysis was performed to provide

energy-resolved insights into the bonding between Co/Ni atoms and N atoms. Due to the limitation of our computing conditions, BNSs were removed to reduce the size of the calculation models, and the effect of the interaction between BNSs and $Co_xNi_{3-x}(HITP)_2$ on N_2 adsorption was neglected. The adsorption energy (E_{ad}) of N_2 on $Co_xNi_{3-x}(HITP)_2$ was calculated by $E_{ad} = E_{total} - E_{MOF} - E_{N_2}$, where E_{total}, E_{MOF}, and E_{N_2} are the DFT calculation energies of the adsorption systems, MOF and an isolated N_2 molecule, respectively.

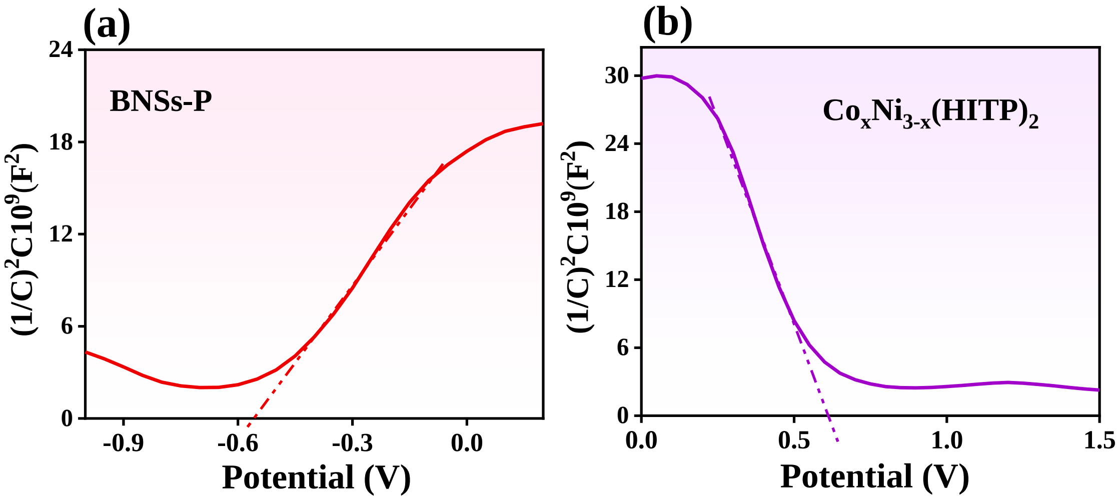

Figure S1. Mott-Schottky curves of (a) BNSs-P and (b) $Co_xNi_{3-x}(HITP)_2$-P catalysts in 0.5 M Na_2SO_4 at −1.0 to 0.0 V vs. Ag/AgCl.

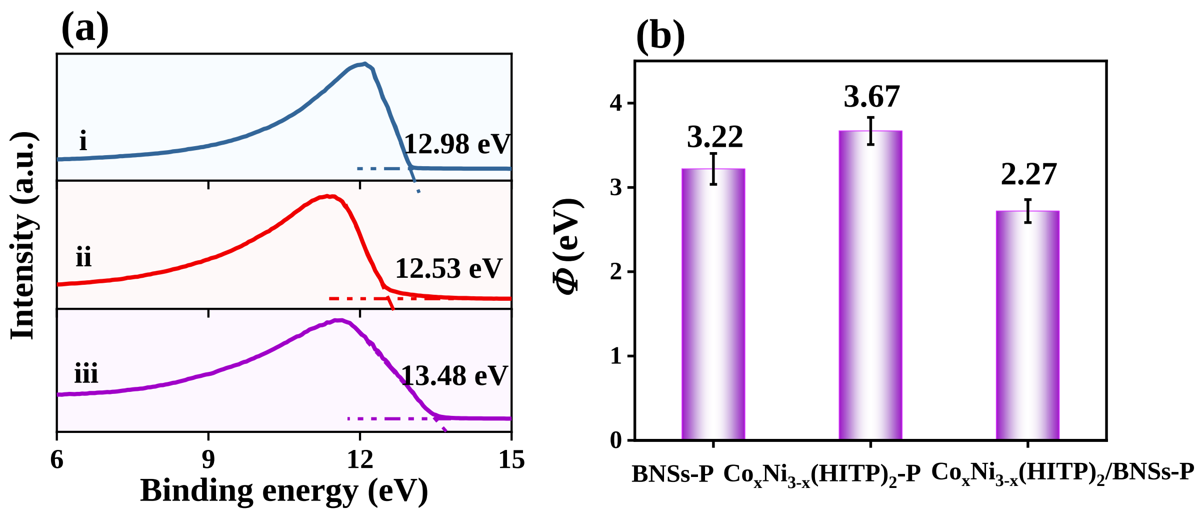

Figure S2. (a) UPS spectra in the normalized secondary electron cutoff energy (E_{cutoff}) regions of (i) BNSs-P, (ii) $Co_xNi_{3-x}(HITP)_2$-P, and (iii) $Co_xNi_{3-x}(HITP)_2$/BNSs-P. (b) Calculated Φ stemming from UPS spectra of BNSs-P, $Co_xNi_{3-x}(HITP)_2$-P, and $Co_xNi_{3-x}(HITP)_2$/BNSs-P.

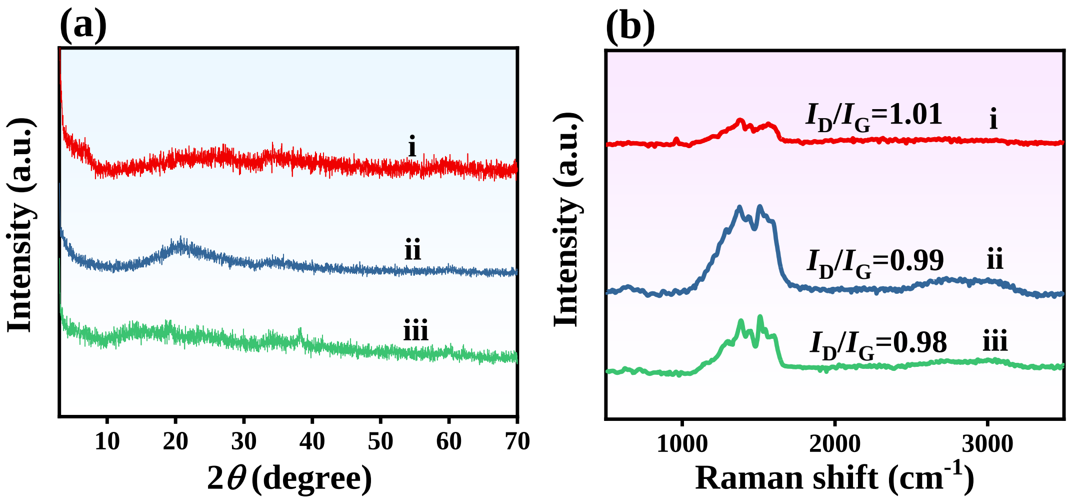

Figure S3. (a) PXRD patterns and (b) Raman spectra of (i) $Co_xCu_{3-x}(HITP)_2$, (ii) $Co_xFe_{3-x}(HITP)_2$ and (iii) $Co_xMn_{3-x}(HITP)_2$. The PXRD patterns (Figure S3a) of $Co_xCu_{3-x}(HITP)_2$, $Co_xFe_{3-x}(HITP)_2$ and $Co_xMn_{3-x}(HITP)_2$ exhibit poor crystallinity, revealing the presence of adequate defects in the prepared MOFs. It also can be proved by Raman spectra of $Co_xCu_{3-x}(HITP)_2$, $Co_xFe_{3-x}(HITP)_2$ and $Co_xMn_{3-x}(HITP)_2$, in which the two strong peaks at 1386 and 1585 cm^{-1} are attributed to the D-band of disordered carbon and G-band of ordered sp^2-carbon, respectively (Figure S3b). The I_D/I_G values of the three bimetallic MOFs are close.

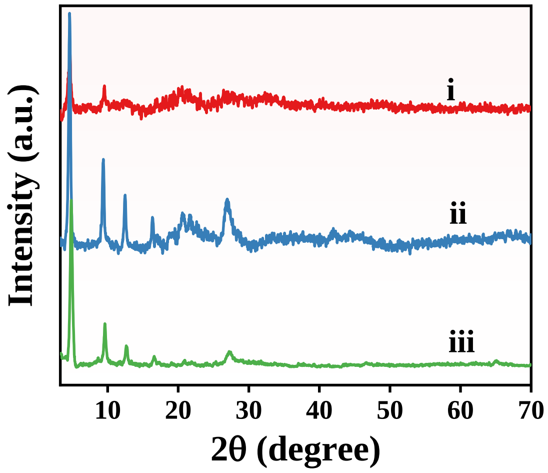

Figure S4. PXRD patterns of (i) $Co_3(HITP)_2$, (ii) $Ni_3(HITP)_2$ and (iii) $Co_xNi_{3-x}(HITP)_2$. The PXRD patterns of $Co_3(HITP)_2$ and $Ni_3(HITP)_2$ show the diffraction peaks at 2θ = 4.6°, 9.5°, 12.6°, and 26.8°, corresponding to the (001), (200), (100) and (220) crystalline planes,[5] respectively. As compared with the PXRD pattern of $Co_3(HITP)_2$, the (001) peak of $Co_xNi_{3-x}(HITP)_2$ shifts to a higher position (2θ = 4.9°), due to the introduction of Ni into $Co_3(HITP)_2$ network.

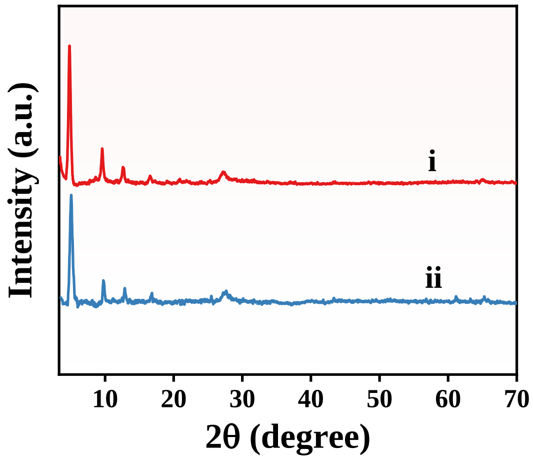

Figure S5. PXRD patterns of $Co_xNi_{3-x}(HITP)_2$ (i) before and (ii) after 24 h 0.1 M HCl. To further investigate the stability of $Co_xNi_{3-x}(HITP)_2$ in 0.1 M HCl, the PXRD patterns of $Co_xNi_{3-x}(HITP)_2$ before and after immersing 0.1 M HCl for 24 h were conducted. No substantial change is observed in the PXRD pattern of $Co_xNi_{3-x}(HITP)_2$, demonstrating its good stability in acidic medium.

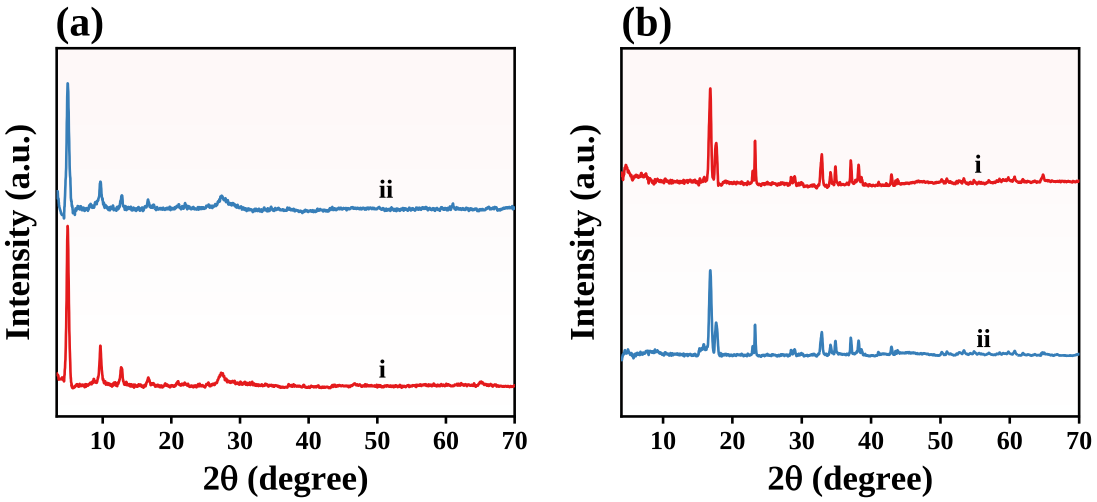

Figure S6. (a) PXRD patterns of (i) $Co_xNi_{3-x}(HITP)_2$ and (ii) $Co_xNi_{3-x}(HITP)_2$-P. (b) PXRD patterns of (i) BNSs and (ii) BNSs-P.

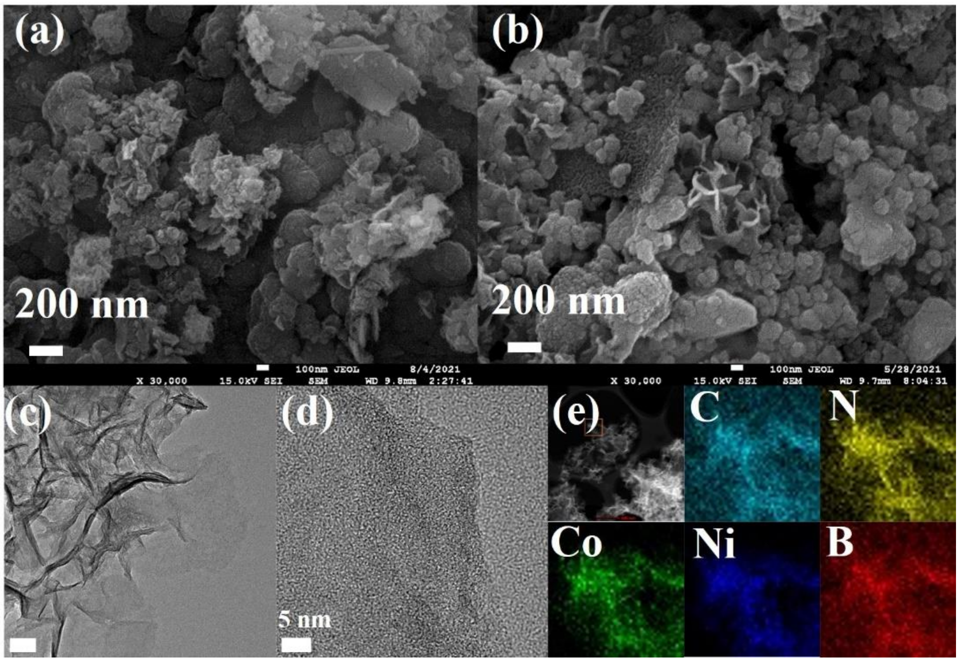

Figure S7. High-magnification FE-SEM images of (a) $Co_xNi_{3-x}(HITP)_2$/BNSs and (b) $Co_xNi_{3-x}(HITP)_2$/BNSs-P. (c, d) TEM image and (e) elemental mapping of Co, Ni, C, N and O for $Co_xNi_{3-x}(HITP)_2$/BNSs.

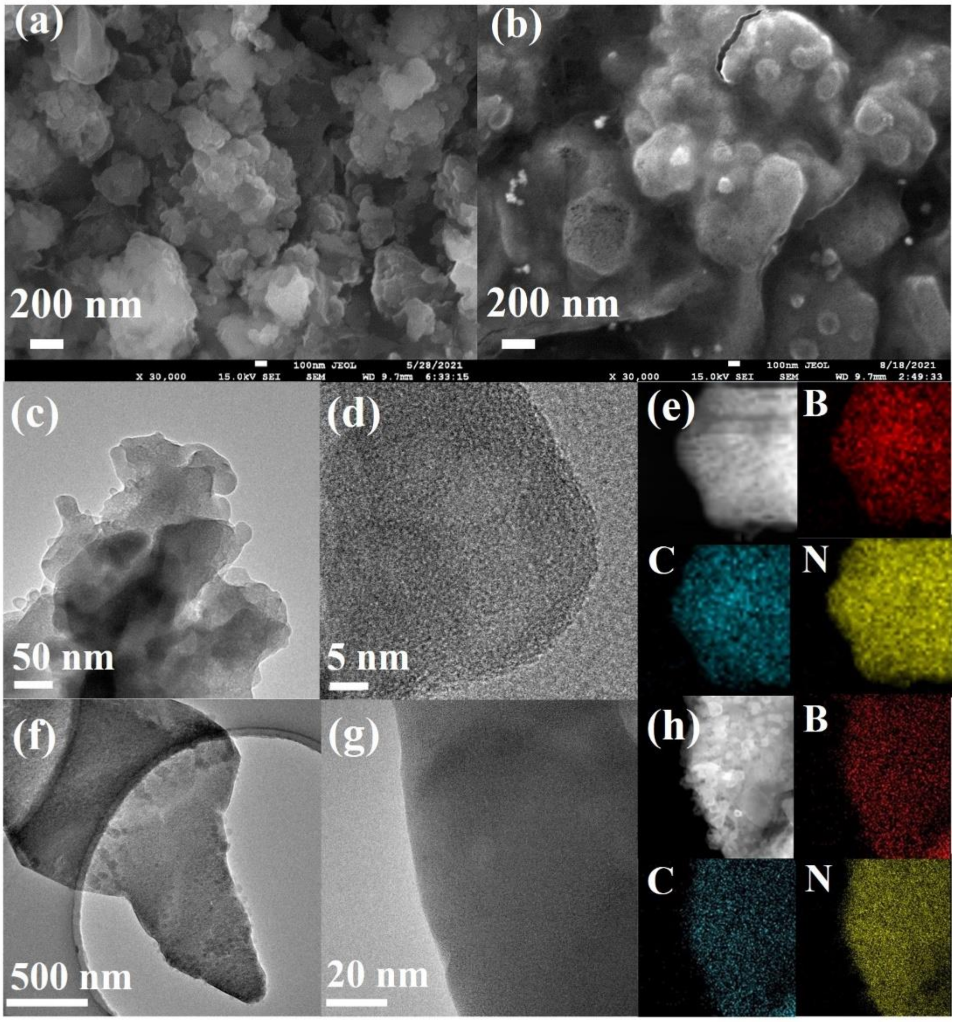

Figure S8. (a, b) Low- and high-magnification SEM images and (c, d) TEM and high-resolution TEM images of BNSs and BNSs-P.

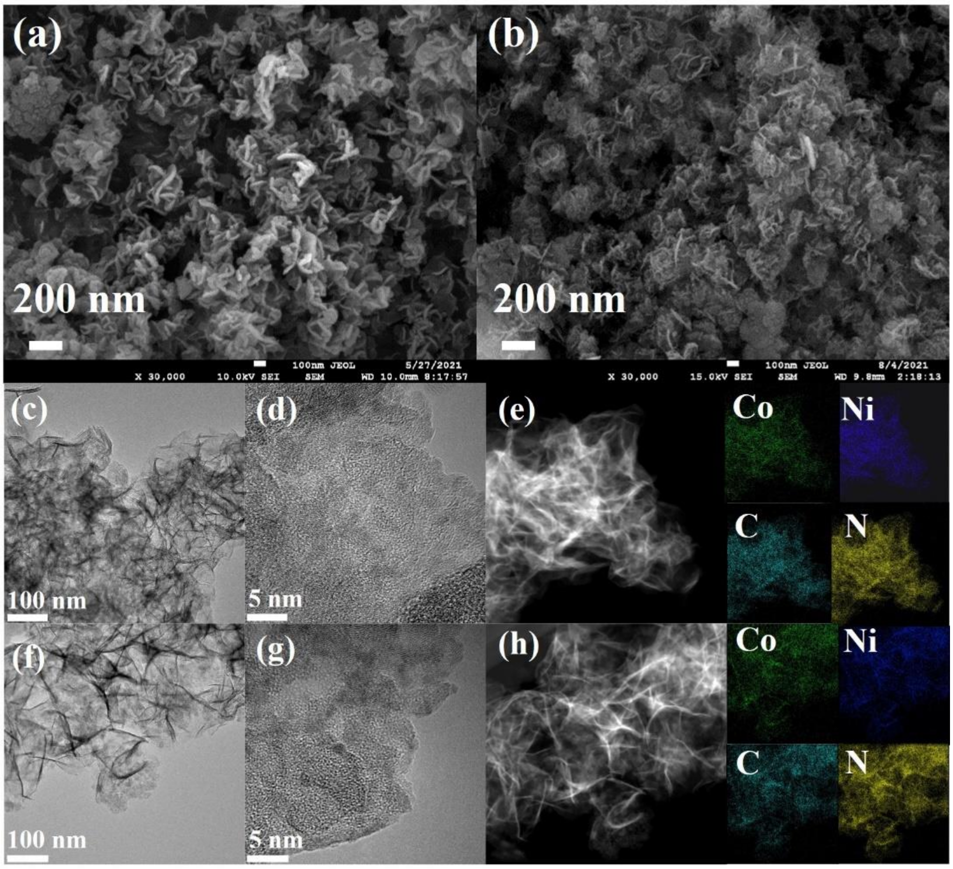

Figure S9. (a, b) Low- and high-magnification SEM images and (c, d) TEM and high-resolution TEM images of $Co_xNi_{3-x}(HITP)_2$ and $Co_xNi_{3-x}(HITP)_2$-P.

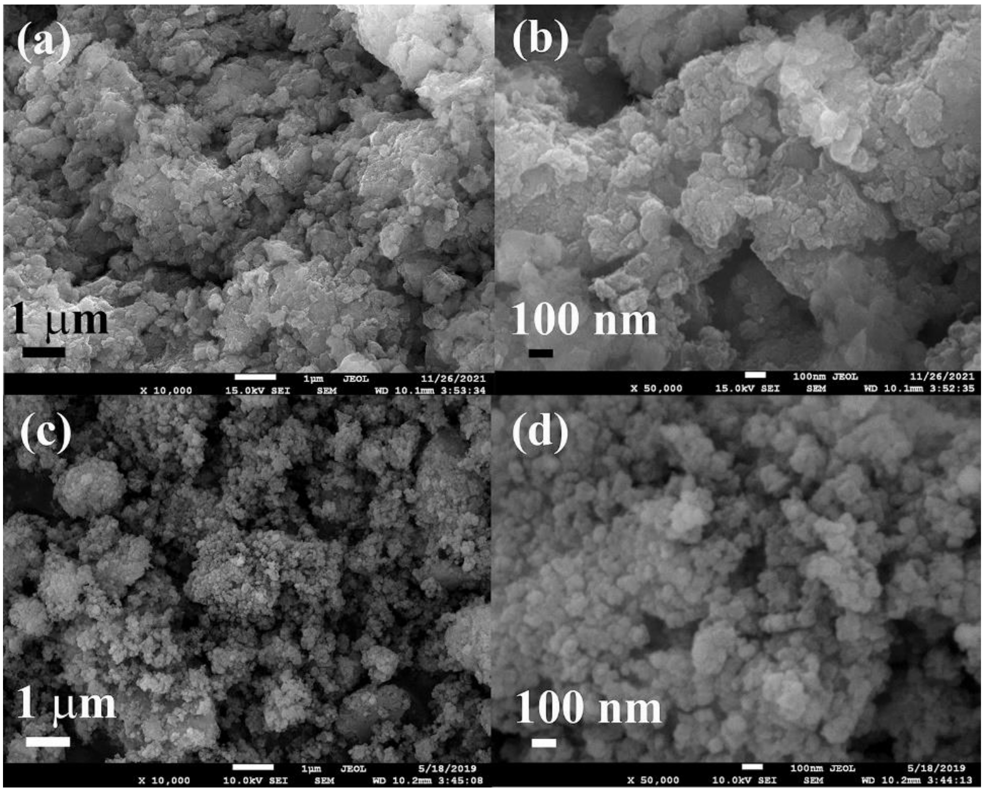

Figure S10. Low- and high-magnification SEM images of (a, b) $Co_3(HITP)_2$ and (c, d) $Ni_3(HITP)_2$. The SEM images show that the monometallic $Co_3(HITP)_2$ and $Ni_3(HITP)_2$ comprise large amounts of irregular nanosheets, which are stacked to large particles.

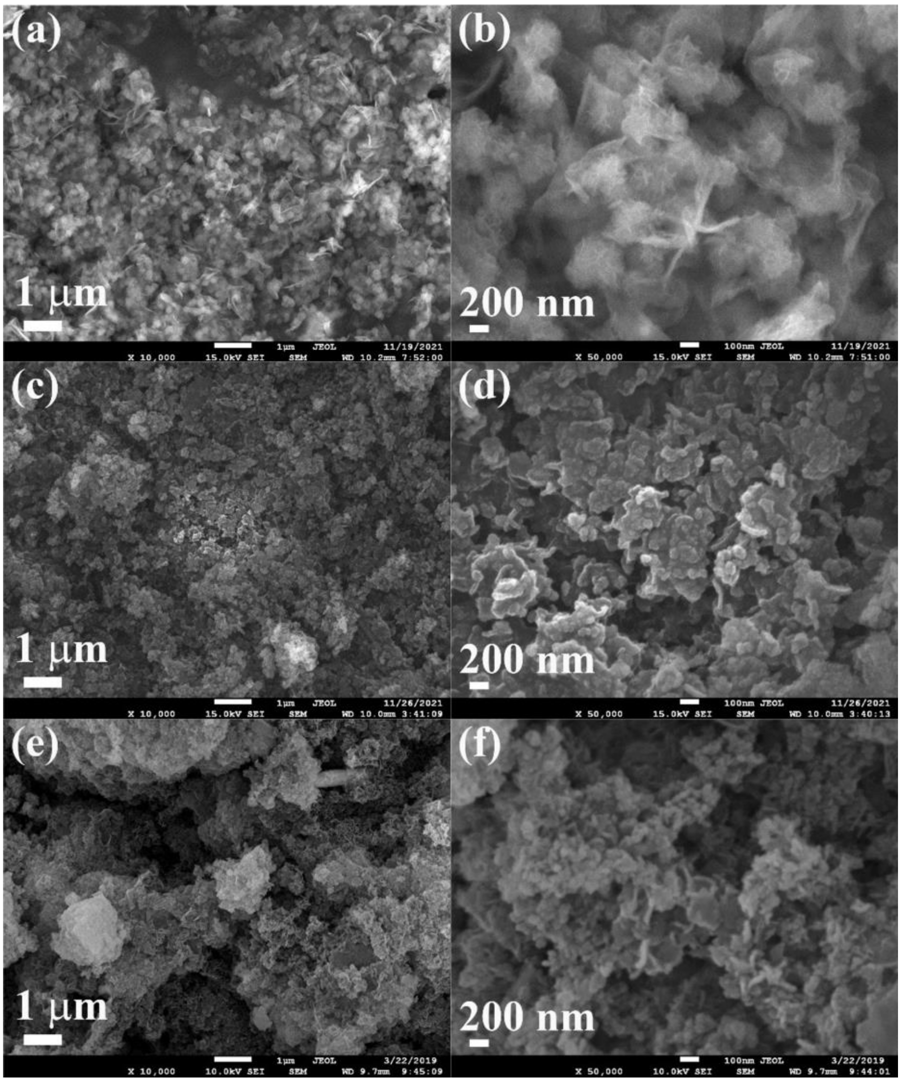

Figure S11. Low- and high-magnification SEM images of (a, b) $Co_xCu_{3-x}(HITP)_2$, (c, d) $Co_xFe_{3-x}(HITP)_2$ and (e, f) $Co_xMn_{3-x}(HITP)_2$. The low-magnification FE-SEM images of diverse bimetallic $Co_xM_{3-x}(HITP)_2$ (M = Cu, Fe and Mn) show nanoflower-like shape, which is composed of irregular nanosheets.

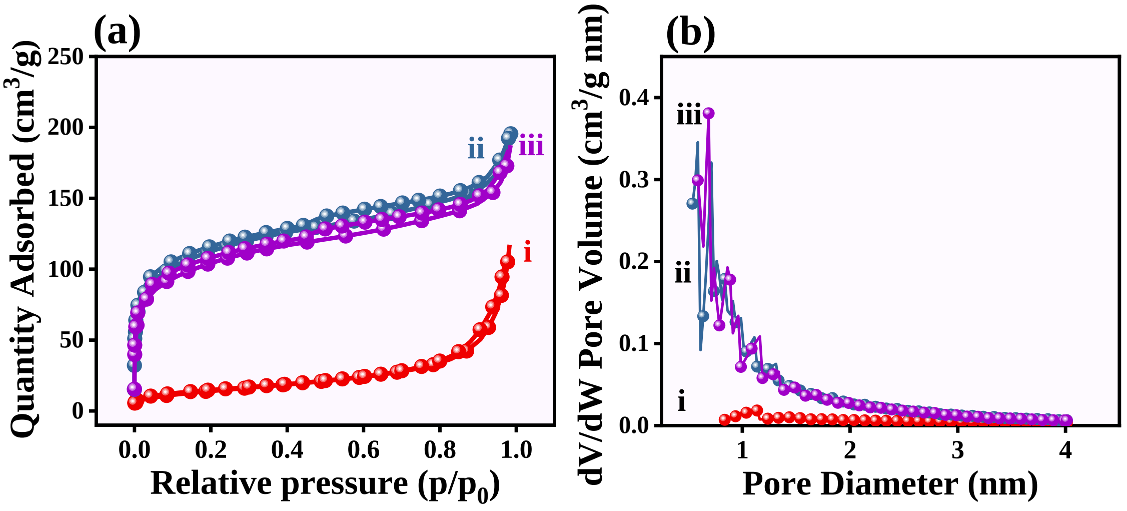

Figure S12. (a) Nitrogen adsorption-desorption isotherm and (b) HK pore size patterns of (i) BNSs, (ii) $Co_xNi_{3-x}(HITP)_2$ and (iii) $Co_xNi_{3-x}(HITP)_2$/BNSs.

Table S1. Summary of surface areas, pore volumes and mean pore sizes of Co_xNi_{3-x}(HITP), BNSs, and Co_xNi_{3-x}(HITP)/BNSs.

**Catalysts**	**BET surface area (m^2 g^{-1})**	**V (cm^3 g^{-1})**	**mean pore size (Å)**
$Co_xNi_{3-x}(HITP)_2$	398.7	0.3024	7.1
BNSs	49.8	0.1813	9.2
Co_xNi_{3-x}(HITP)/BNSs	368.2	0.2899	6.8

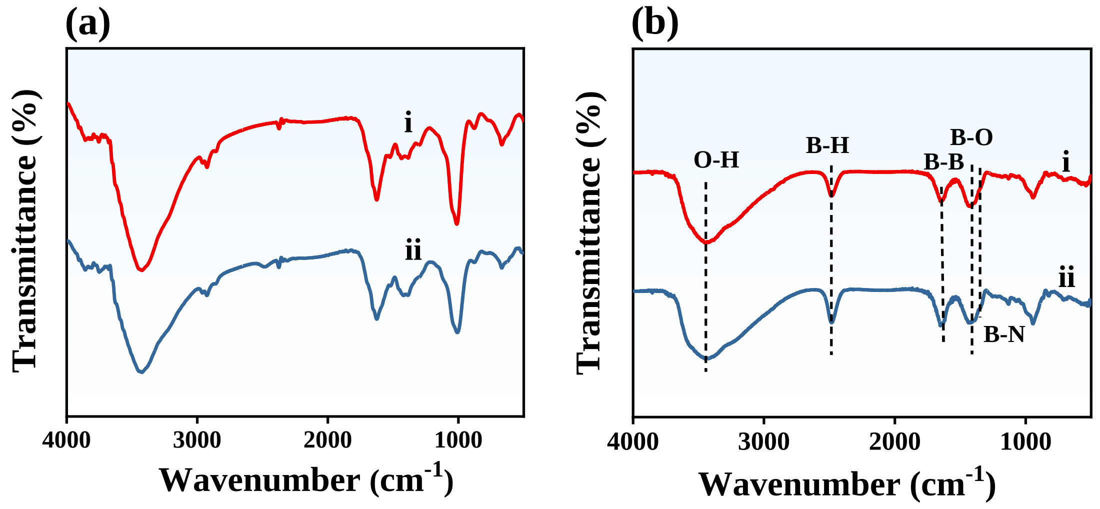

Figure S13. (a) FT-IR spectra of (i) BNSs and (ii) BNSs-P. (b) FT-IR spectra of (i) $Co_xNi_{3-x}(HITP)_2$ and (ii) $Co_xNi_{3-x}(HITP)_2$-P.

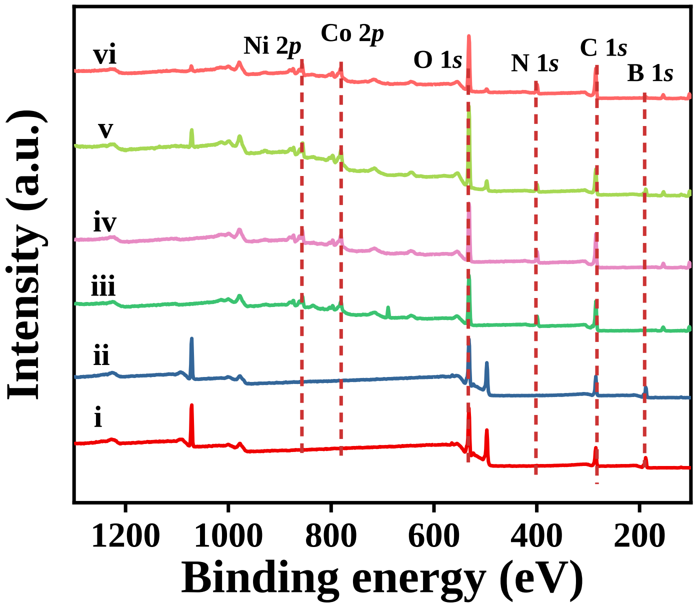

Figure S14. XPS survey scan spectra of (i) BNSs, (ii) BNSs-P, (iii) $Co_xNi_{3-x}(HITP)_2$, (iv) $Co_xNi_{3-x}(HITP)_2$-P, (v) $Co_xNi_{3-x}(HITP)_2$/BNSs and (vi) $Co_xNi_{3-x}(HITP)_2$/BNSs-P. $Co_xNi_{3-x}(HITP)_2$ shows the signals of Co 2*p* (782.1 eV), Ni 2*p* (858.7 eV), C 1*s* (282.7 eV), O 1*s* (531.7 eV), and N 1*s* (399.8 eV), while BNSs exhibit the B 1*s* (188.6 eV), C 1*s*, and O 1*s*. The $Co_xNi_{3-x}(HITP)_2$/BNSs heterostructure combines the signals of all elements.

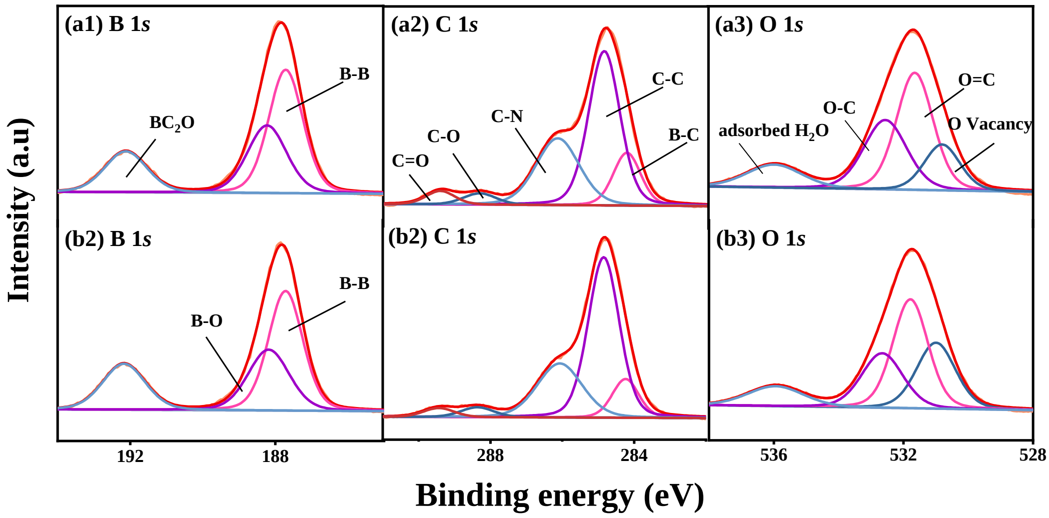

Figure S15. High-resolution XPS spectra of B 1*s*, N 1*s* and O 1*s* of (a1, b1, c1) BNSs and (a2, b2, c2) BNSs-P. The B 1*s* XPS spectrum of BNSs-P can be fitted out to three peaks at the binding energies (BEs) of 187.7, 188.7 and 191.6 eV, indicating the generation of B atoms with different chemical environments. It indicates that BNSs have tunable electronic structure.[6] The presence of apparent B-O group suggests the partial oxidation of BNSs during the preparation procedure. As compared, the relative content of B-O of BNSs-P (23%) is larger than that of BNSs-P (17%), due to the reaction of boron atoms activated by plasma irradiation with oxygen-related species derived from water, forming B-O bonds. Further, the C 1*s* XPS spectra of BNSs before and after the plasma irradiation can be de deconvoluted to five parts, including C-B (284.3 eV), C-C (284.8 eV), C-O (286.2 eV), C=O (288.3 eV), and COO (289.4 eV). Amongst, C-C group is dominant in the C component, while no significant change can be observed for BNSs before and after the plasma irradiation. In addition, the O 1*s* spectra of BNSs-P can be fitted out to four peaks at 530.7, 531.6, 532.5 and 535.9 eV corresponding to oxygen vacancies, C-O, C=O and adsorbed H_2O, respectively. Compared with the pristine BNSs, the relative content of oxygen vacancies of BNSs-P increases from 18% to 26%, which is consistent with the EPR results.

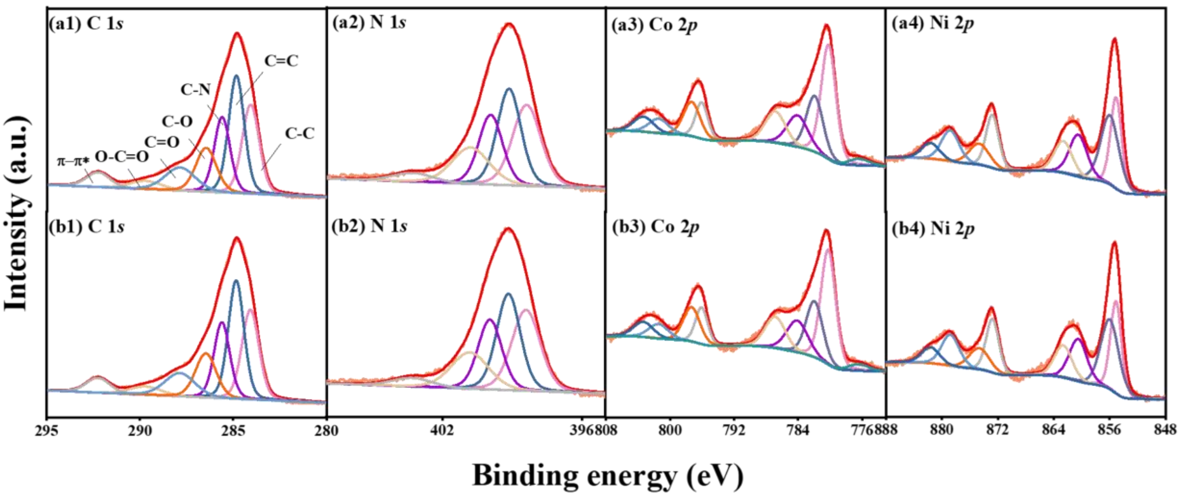

Figure S16. High-resolution XPS spectra of C 1*s*, N 1*s*, Co 2*p* and Ni 2*p* of (a1, a2, a3, a4) $Co_xNi_{3-x}(HITP)_2$ and (b1, b2, b3, b4) $Co_xNi_{3-x}(HITP)_2$-P. The Co 2*p* XPS spectra of $Co_xNi_{3-x}(HITP)_2$ and $Co_xNi_{3-x}(HITP)_2$-P are similar to that of $Co_xNi_{3-x}(HITP)_2$/BNSs-P, comprising the mixed Co^{2+}/Co^{3+} valences (Figure S15a3). Their Ni XPS spectra also illustrate the analogous results with the $Co_xNi_{3-x}(HITP)_2$/BNSs junction, showing the combination of Ni^{2+} and Ni^{3+} species (Figure S15a4). Further, the N 1*s* XPS spectra (Figure S15a2) are composed of four groups of pyridinic N (398.8 eV), pyrrolic N (399.7 eV), graphitic N (400.7 eV), and oxidized N (401.9 eV). The C 1*s* XPS spectra is composed of C-C, C-N, C-O, C=O, and COO (Figure S15a1). Similarly, no substantial difference can be found in $Co_xNi_{3-x}(HITP)_2$ and $Co_xNi_{3-x}(HITP)_2$-P.

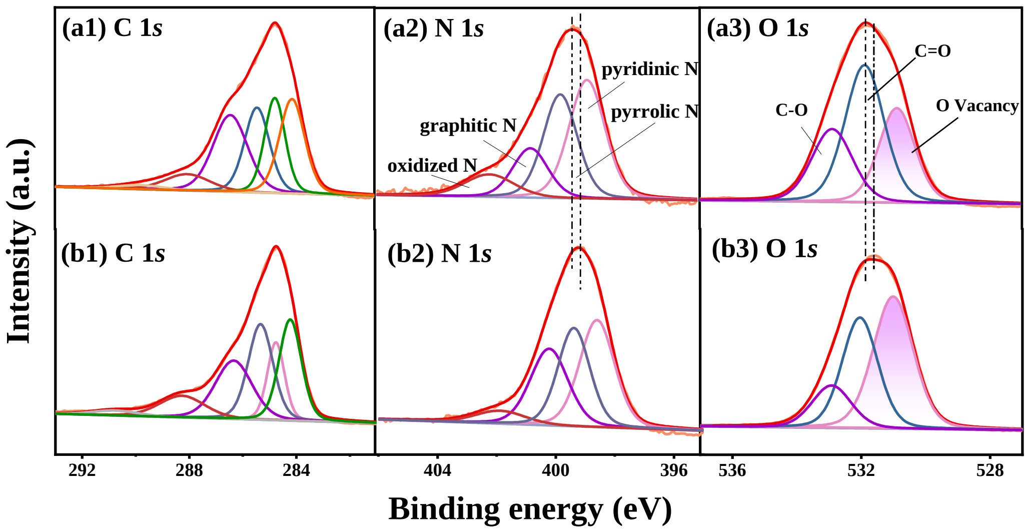

Figure S17. High-resolution C 1*s*, N 1*s* and O 1*s* of (a1, a2, a3) $Co_xNi_{3-x}(HITP)_2$/BNSs and (b1, b2, b3) $Co_xNi_{3-x}(HITP)_2$/BNSs-P.

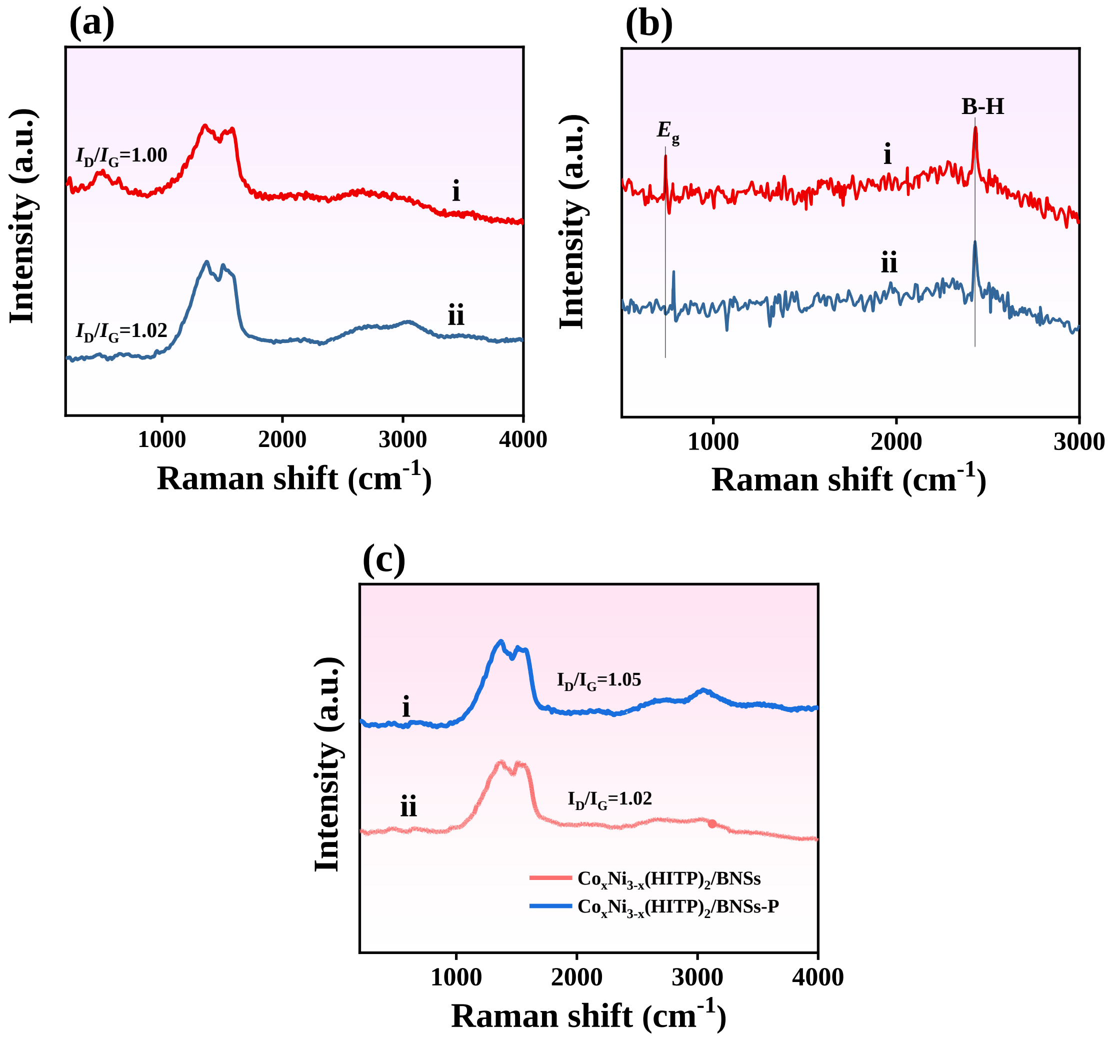

Figure S18. Raman spectra of (a) BNSs, (b) $Co_xNi_{3-x}(HITP)_2$, (c) $Co_xNi_{3-x}(HITP)_2$/BNSs before (i) and after plasma modification (ii).

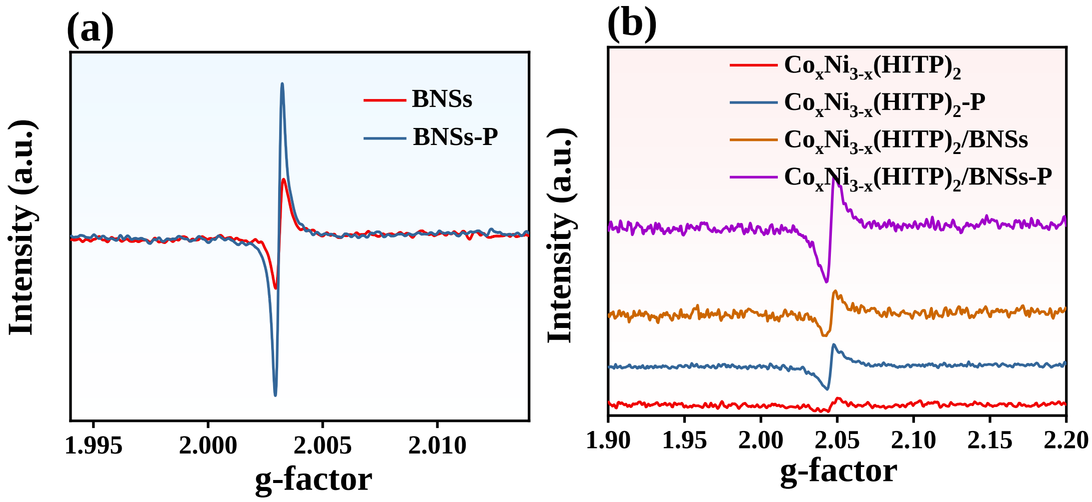

Figure S19. EPR spectra of (a) BNSs, (b) $Co_xNi_{3-x}(HITP)_2$ and $Co_xNi_{3-x}(HITP)_2$/BNSs before and after treated by plasma irradiation.

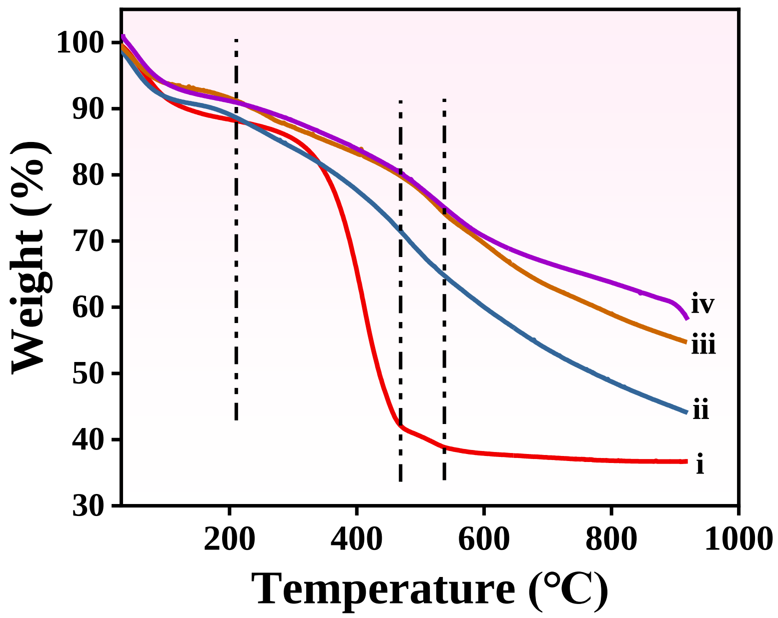

Figure S20. TGA curves of (i) $Co_xNi_{3-x}(HITP)_2$, (ii) $Co_xNi_{3-x}(HITP)_2$-P, (iii) $Co_xNi_{3-x}(HITP)_2$/BNSs, and (iv) $Co_xNi_{3-x}(HITP)_2$/BNSs-P.

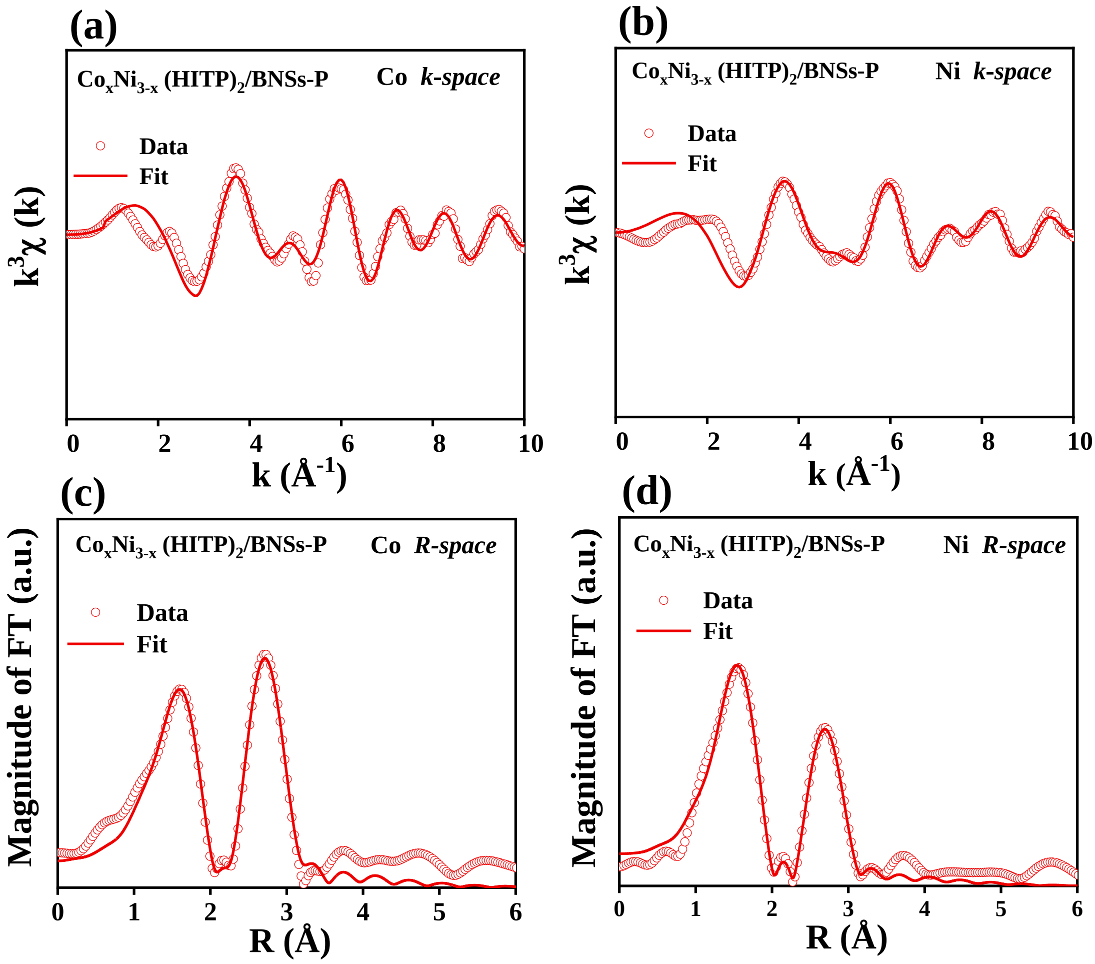

Figure S21. (a) Co k space and (b) Ni k space fitting curves of $Co_xNi_{3-x}(HITP)_2$/BNSs-P. (c, d) The corresponding K-edge Fourier-transform EXAFS of $Co_xNi_{3-x}(HITP)_2$/BNSs-P.

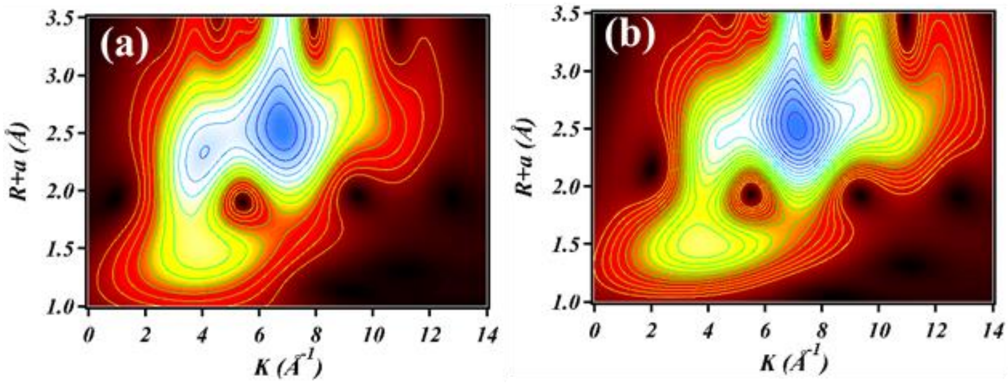

Figure S22. Wavelet transform images of (a) CoO and (b) NiO.

Table S2. Coordination number of Co and Ni extracted from the EXAFS fitting.

	Path	N	ΔE (eV)	100R (Å)	1000σ^2 (Å^2)	R-factor
Co foil	Co-Co	12*	2.49±0.01	0.0061±0.0004	-5.9±0.595	0.004
CoPc	Co-N	4.0±0.7	1.89±0.01	0.002±0.002	3.50±3.23	0.018
Co_xNi_{3-}	Co-N	3.38±0.5	2.06±0.01	0.008±0.002	-1.57±1.66	0.007
$_x(HITP)_2$/BNSs-P	Co-Co	5.14±0.9	3.13±0.01	0.009±0.001	2.43±1.56	
Ni Foil	Ni-Ni	12*	2.48±0.01	0.005±0.0002	0.71±0.02	0.002
NiPc	Ni-N	3.7±0.8	1.88±0.01	0.0016±0.002	-4.02±3.62	0.016
Co_xNi_{3-}	Ni-N	3.98±0.4	2.06±0.01	0.008±0.001	-6.31±1.02	0.006
$_x(HITP)_2$/BNSs-P	Ni-Ni	3.16±0.5	3.13±0.01	0.009±0.001	-0.87±1.54	

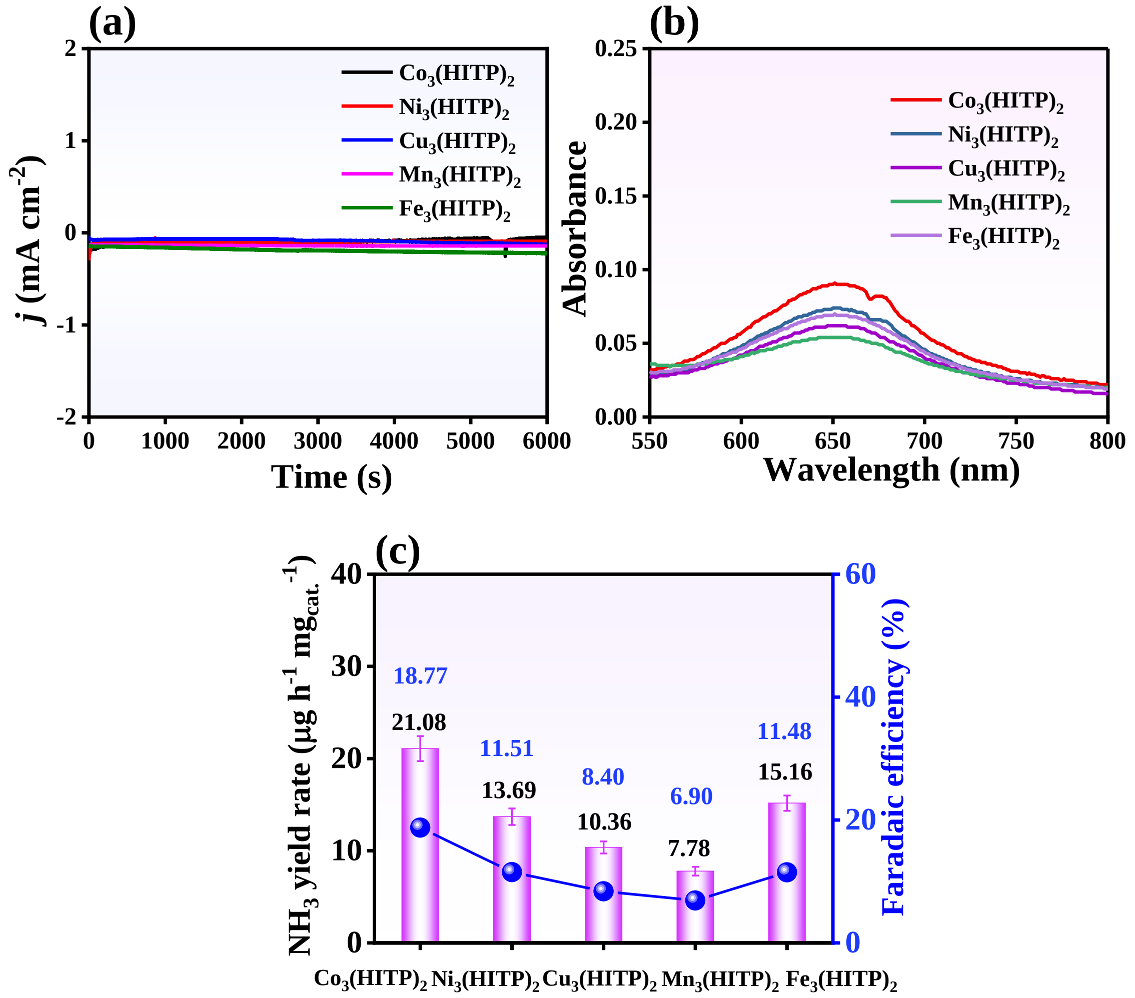

Figure S23. (a) Chronoamperometry curves of the $Co_3(HITP)_2$, $Ni_3(HITP)_2$ $Cu_3(HITP)_2$, $Mn_3(HITP)_2$ and $Fe_3(HITP)_2$. (b) UV-vis absorption spectra of $Cu_3(HITP)_2$, $Mn_3(HITP)_2$ and $Fe_3(HITP)_2$ in N_2 and Ar saturated 0.1 M HCl electrolyte at -0.4 V. (c) The NH_3 yield rate and FE of $Co_3(HITP)_2$, $Ni_3(HITP)_2$, $Cu_3(HITP)_2$, $Mn_3(HITP)_2$ and $Fe_3(HITP)_2$.

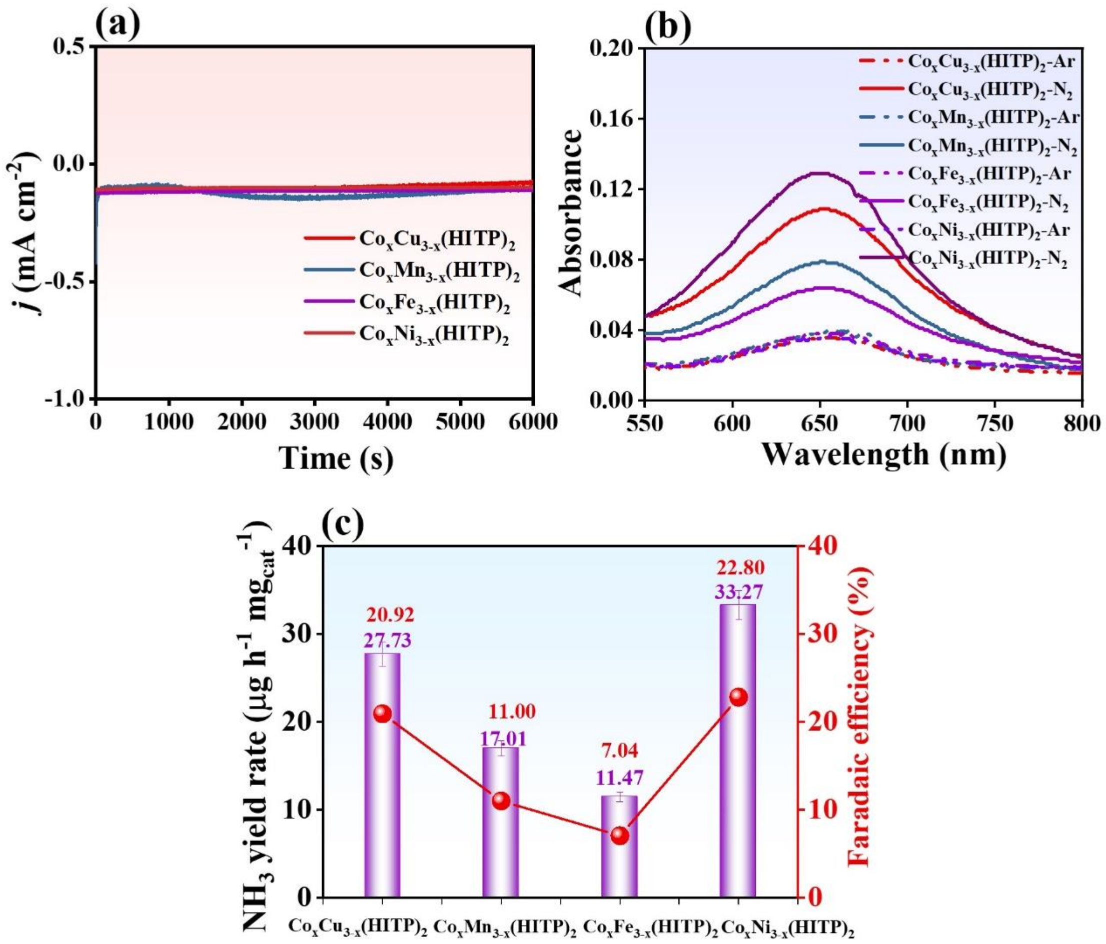

Figure S24. (a) Chronoamperometry curves of $Co_xCu_{3-x}(HITP)_2$, $Co_xMn_{3-x}(HITP)_2$ and $Co_xFe_{3-x}(HITP)_2$. (b) UV-vis absorption spectra of $Co_xCu_{3-x}(HITP)_2$, $Co_xMn_{3-x}(HITP)_2$ and $Co_xFe_{3-x}(HITP)_2$ in N_2 and Ar saturated 0.1 M HCl electrolyte at -0.4 V. (c) The NH_3 yield rate and Faradaic efficiency of $Co_xCu_{3-x}(HITP)_2$, $Co_xMn_{3-x}(HITP)_2$, $Co_xFe_{3-x}(HITP)_2$ and $Co_xNi_{3-x}(HITP)_2$.

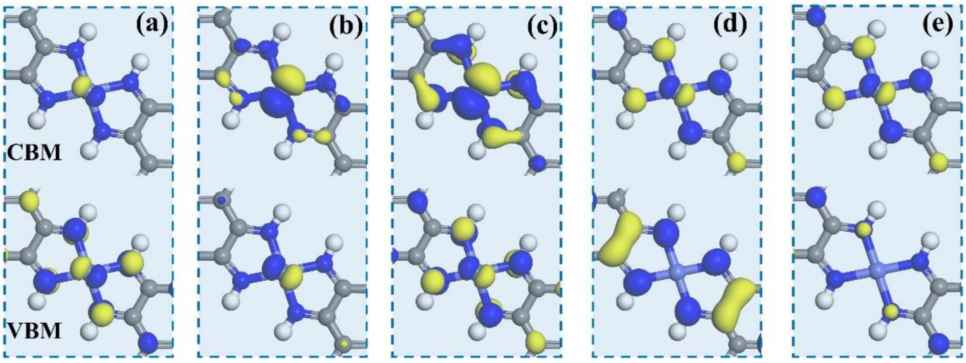

Figure S25. The CBM and VBM distribution diagrams of (a) $Co_3(HITP)_2$, (b) $Co_xCu_{3-x}(HITP)_2$, (c) $Co_xFe_{3-x}(HITP)_2$, (d) $Co_xMn_{3-x}(HITP)_2$, and (e) $Co_xNi_{3-x}(HITP)_2$.

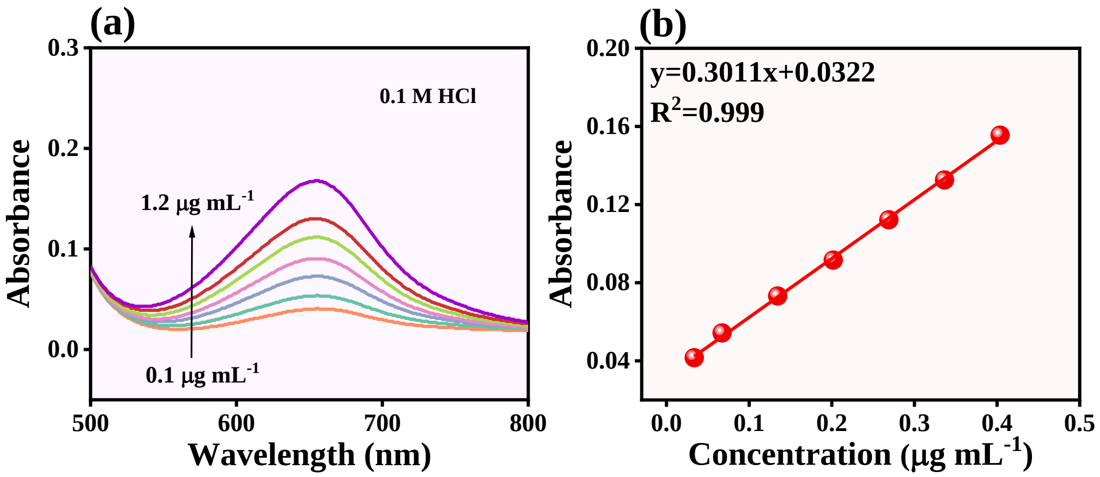

Figure S26. (a) UV-vis absorption curves of indophenol assays with NH_4Cl after incubating for 2 h and (b) corresponding calibration curve for calculation of NH_3 concentrations.

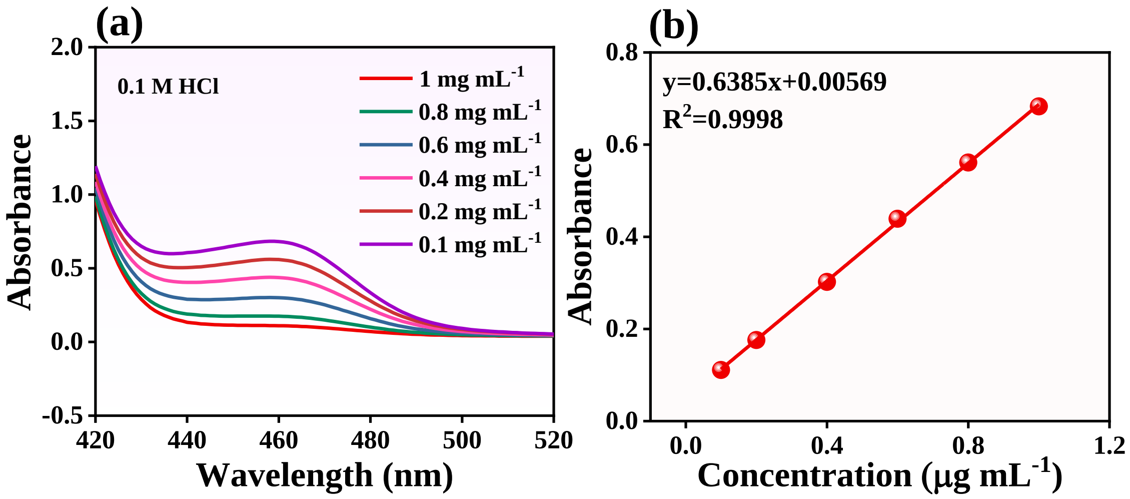

Figure S27. Calibration of the estimation method for subsequent hydrazine quantification. (a) UV-vis absorption spectra and (b) the calibration curve of colorimetric N_2H_4 assay using the Watt-Chrisp method.

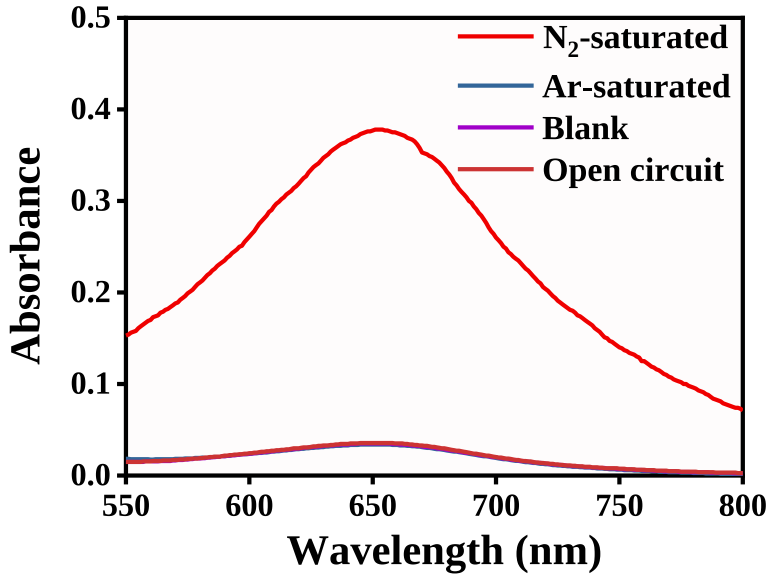

Figure S28. UV-vis absorption spectra of $Co_xNi_{3-x}(HITP)_2$/BNSs-P in N_2 or Ar saturated 0.1 M HCl electrolyte with and without applied potential (-0.4 V *vs.* RHE).

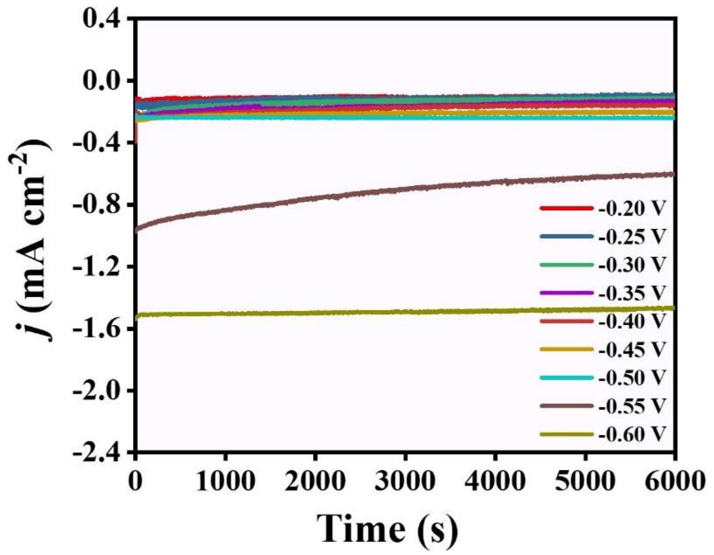

Figure S29. Chronoamperometry curves of the $Co_xNi_{3-x}(HITP)_2$/BNSs-electrode at various potentials vs. RHE.

Table S3. Comparison of the eNRR activity of reported electrocatalysts in 0.1 M HCl.

Catalysts	Electrolyte	NH_3 yield ($\mu g\ h^{-1}\ mg_{cat.}{}^{1}$)	FE (%)	Ref.
Co_4N/Co_2C@rGO	0.1 M HCl	24.12	24.97	[7]
MoFe-PC	0.1 M HCl	34.23	16.83	[8]
MoB_2	0.05 M H_2SO_4	40.94	30.84	[9]
B_4C	0.1 M HCl	26.57	16.7	[10]
SA-Mo/NPC	0.1 M HCl	31.5	14.6	[11]
Mo-Co/NC	0.1 M Na_2SO4	89.8	13.5	[12]
FL-BP NSs	0.1 M HCl	31.37	5.07	[13]
Zn-Co_3O_4	0.1 M HCl	22.71	11.9	[14]
MoO_{3-x}/MXene	0.1 M Na_2SO_4	95.8	22.3	[15]
FePc-pz	0.01 M H_2SO_4	33.6	31.9	[16]
$Co_xNi_{3-x}(HITP)_3$/BNSs	0.1 M HCl	128.26	52.9	this work

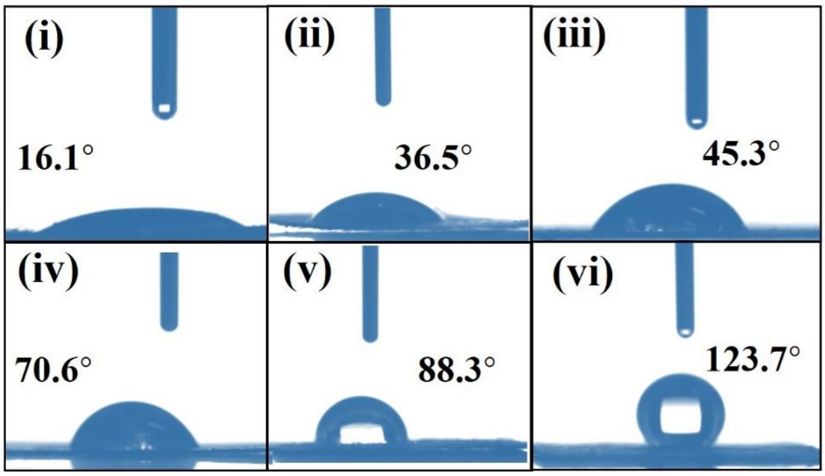

Figure S30. Static water contact angle images of (i) BNSs, (ii) BNSs-P, (iii) $Co_xNi_{3-x}(HITP)_2$, (iv) $Co_xNi_{3-x}(HITP)_2$-P, (v) $Co_xNi_{3-x}(HITP)_2$/BNSs, and (vi) $Co_xNi_{3-x}(HITP)_2$/BNSs-P.

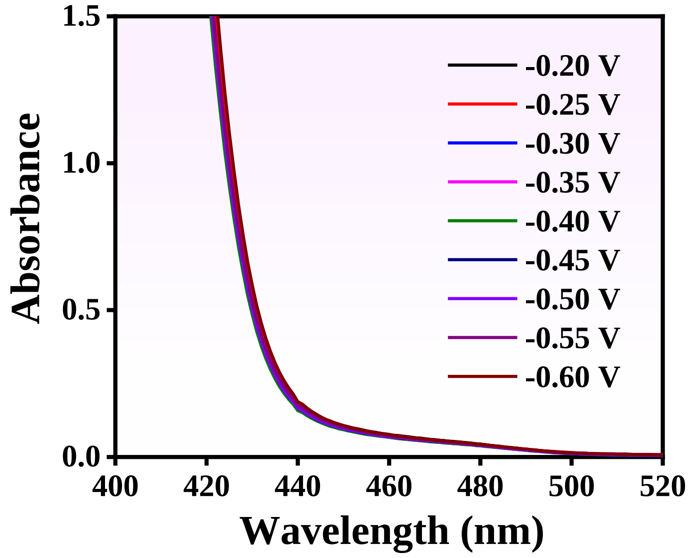

Figure S31. UV-vis absorption spectra of electrolytes at certain potential stained with hydrazine hydrate indicator after 6000 s eNRR electrolysis.

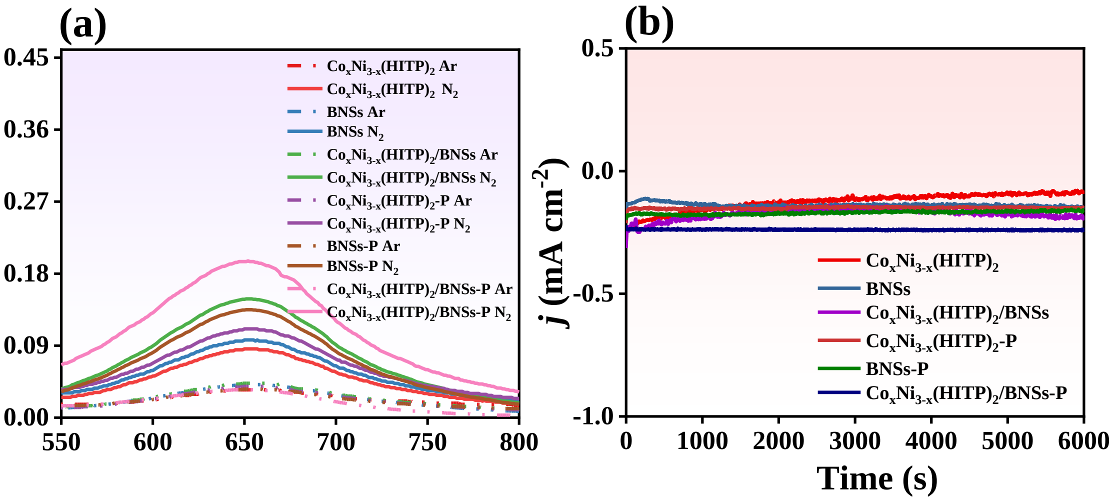

Figure S32 (a) UV-vis absorption spectra of different catalysts in 0.1 M N_2-saturated HCl solution at v-0.5 V vs. RHE. (b) Chronoamperometry curves of different catalysts at -0.5 V vs. RHE.

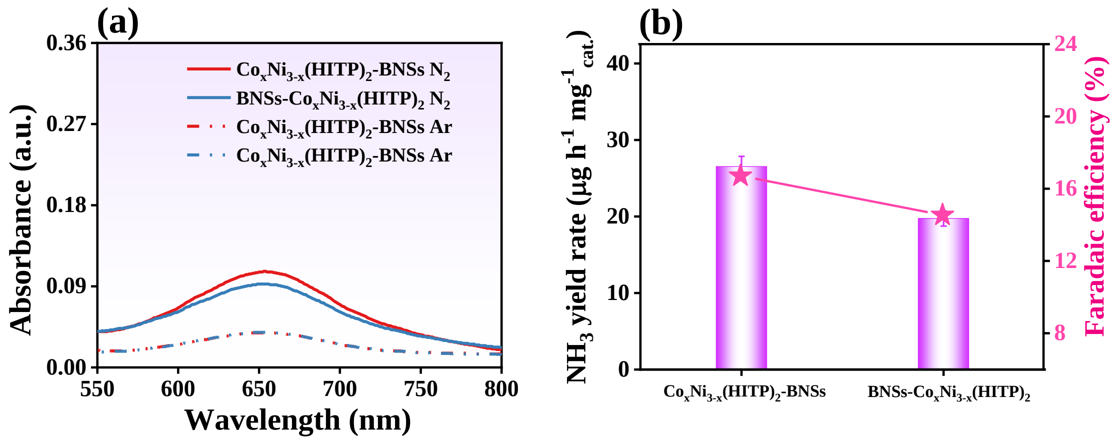

Figure S33 (a) UV-vis absorption spectra of $Co_xNi_{3-x}(HITP)_2$-BNSs and BNSs-$Co_xNi_{3-x}(HITP)_2$ in 0.1 M N_2-saturated HCl solution at various applied potentials. (c) Dependence of NH_3 yield and Faradaic efficiency of $Co_xNi_{3-x}(HITP)_2$-BNSs and BNSs-$Co_xNi_{3-x}(HITP)_2$ at each applied potential in N_2-saturated 0.1 M HCl electrolyte with the NRR measurement time of 6000 s.

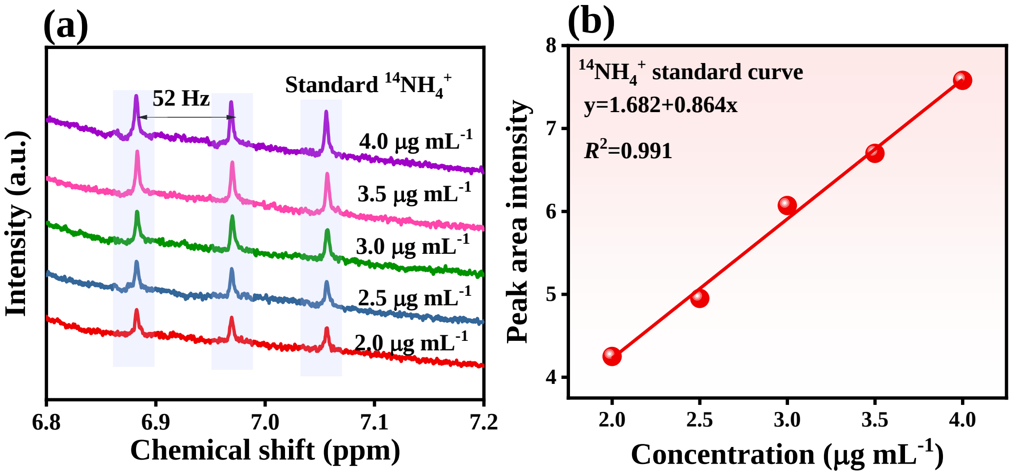

Figure S34. (a) ^{1}H NMR spectra of $^{14}NH_4^+$standard samples with different concentrations and (b) the corresponding calibration curve for the evaluation of $^{14}NH_4^+$ concentration.

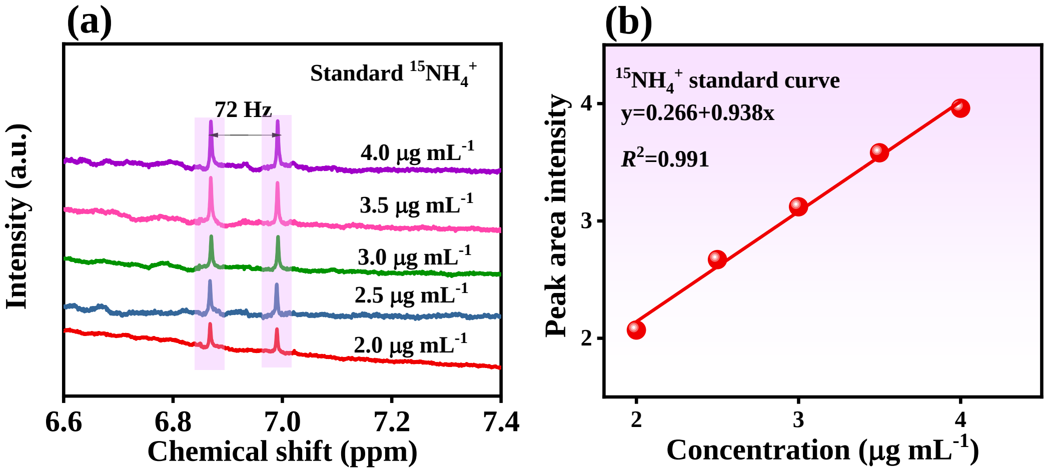

Figure S35. (a) ^{1}H NMR spectra of $^{15}NH_4^+$ standard samples with different concentrations and (b) the corresponding calibration curve for the evaluation of $^{15}NH_4^+$ concentration.

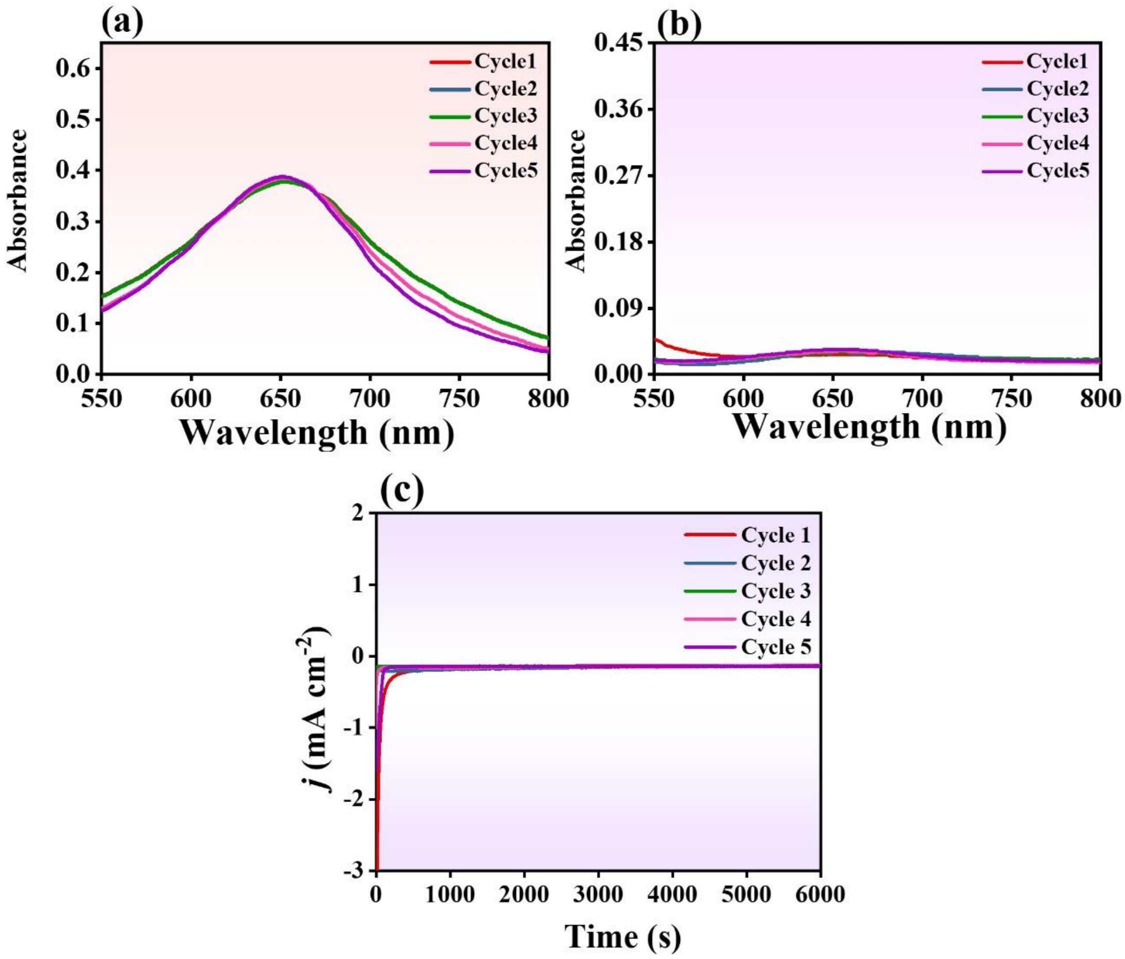

Figure S36. UV-vis absorption spectra of $Co_xNi_{3-x}(HITP)_2$/BNSs-P after cycle test of $Co_xNi_{3-x}(HITP)_2$/BNSs-P in (a) N_2 saturated and (b) Ar saturated 0.1 M HCl electrolyte at -0.4 V. (c) Chronoamperometry curves for each electrolysis cycle of $Co_xNi_{3-x}(HITP)_2$/BNSs-P at -0.4 V.

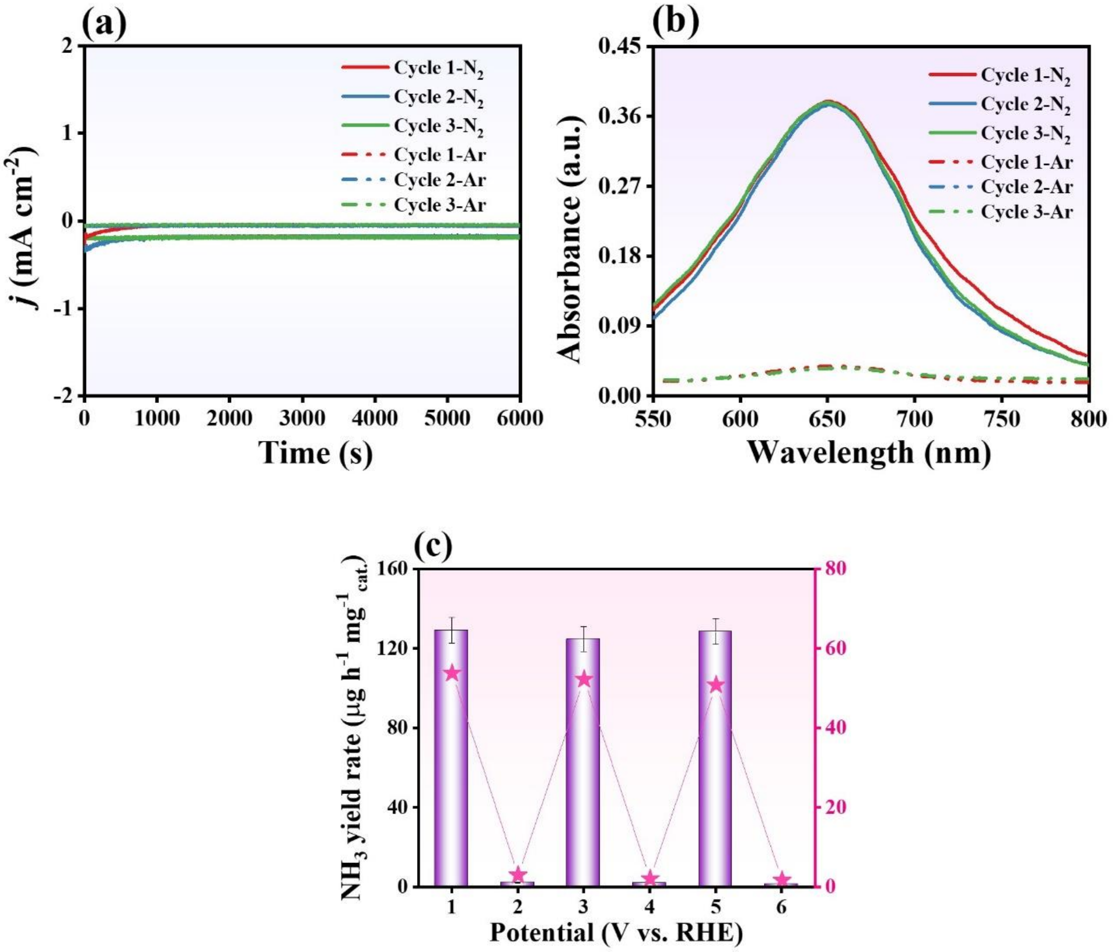

Figure S37. Chronoamperometry curves of the $Co_xNi_{3-x}(HITP)_2$/BNSs-electrode at -0.4 vs RHE. (b) Corresponding UV-vis absorption spectra. (c) Ar/N_2 alternating cycling test of $Co_xNi_{3-x}(HITP)_2$/BNSs-P.

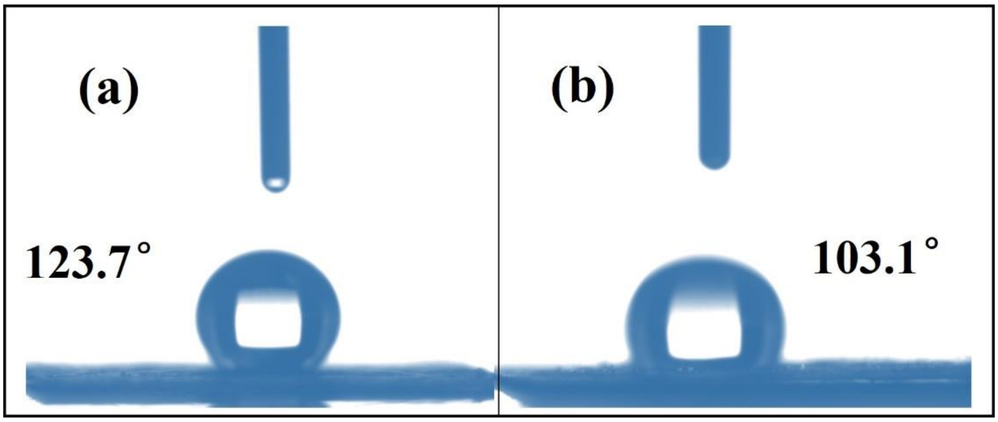

Figure S38. Static water contact angle images of $Co_xNi_{3-x}(HITP)_2$/BNSs-P (a) before and (b) after chronoamperometry tests.

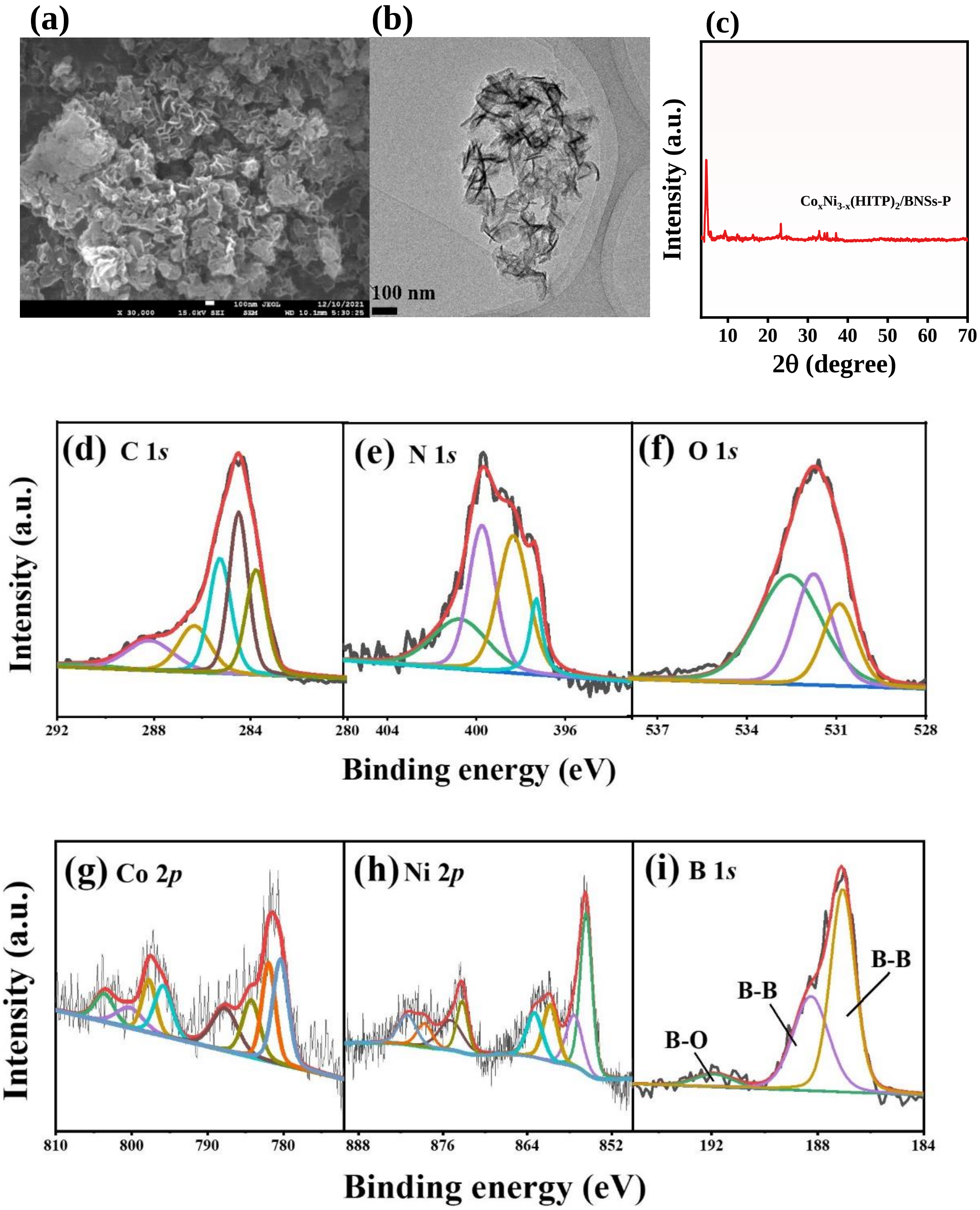

Figure S39. (a) High-magnification SEM image, (b) TEM image and (c) PXRD pattern of the used $Co_xNi_{3-x}(HITP)_2$/BNSs-P. XPS spectra of (d) C 1*s*, (e) N 1*s*, (f) O 1*s*, (g) Co 2*p*, (h) Ni 2*p* and (i) B 1*s* in the used $Co_xNi_{3-x}(HITP)_2$/BNSs-P.

Table S4 Co and Ni of $Co_xNi_{3-x}(HITP)_2$/BNSs-P in 0.1 M HCl

**Time (h)**	**Element (mg L^{-1})**	
	Co	Ni
0	0.000	0.002
3	0.168	0.025
6	0.268	0.036
9	0.288	0.038
12	0.289	0.039
15	0.291	0.037

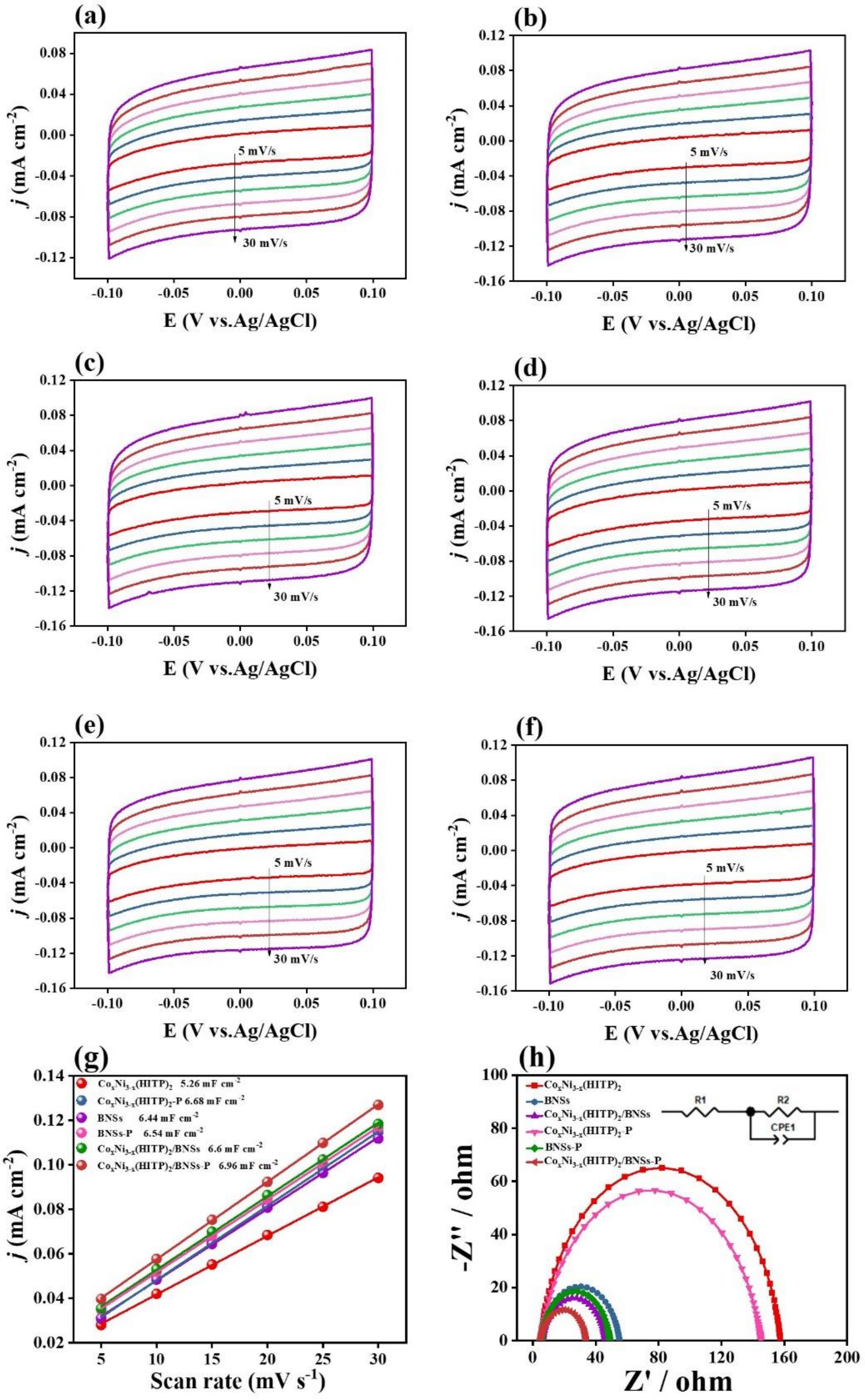

Figure S40. Cyclic voltammetry curves of (a) $Co_xNi_{3-x}(HITP)_2$, (b) $Co_xNi_{3-x}(HITP)_2$-P, (c) BNSs, (d) BNSs-P, (e) $Co_xNi_{3-x}(HITP)_2$/BNSs and (f) $Co_xNi_{3-x}(HITP)_2$/BNSs-P. (g) The corresponding electrochemical double layer capacitances. (h) Nyquist plots at -0.4 V *vs.* RHE (inset: the equivalent circuit).

Table S5. C_{dl} and R_{ct} values of all samples in 0.1 M HCl electrolyte.

Sample	C_{dl} (mF cm^{-2})	R_{ct} (Ω)
$Co_xNi_{3-x}(HITP)_2$	5.26	152
BNSs	6.44	50
$Co_xNi_{3-x}(HITP)_2$/BNSs	6.60	40
$Co_xNi_{3-x}(HITP)_2$-P	6.68	140
BNSs-P	6.54	44
$Co_xNi_{3-x}(HITP)_2$/BNSs-P	6.96	29

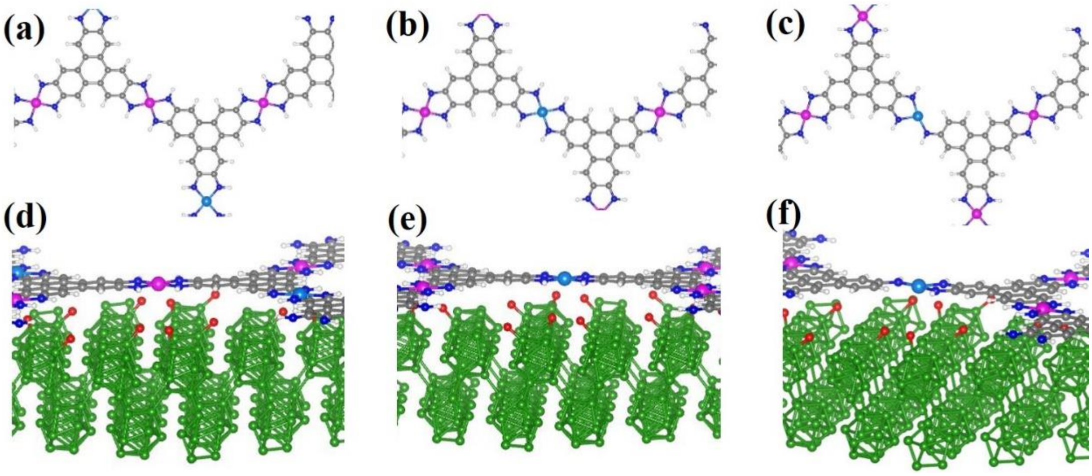

Figure S41. Atomic structure model of (a) Ni-N_4, (b) Co-N_4, (c) Co-N_3, (d) Ni-N_4/BNSs, (e) Co-N_4/BNSs and (f) Co-N_3/BNSs. Atoms: gray spheres for C, blue spheres for N, light blue spheres for Co, pink sphere for Ni, red sphere for O and green sphere for B.

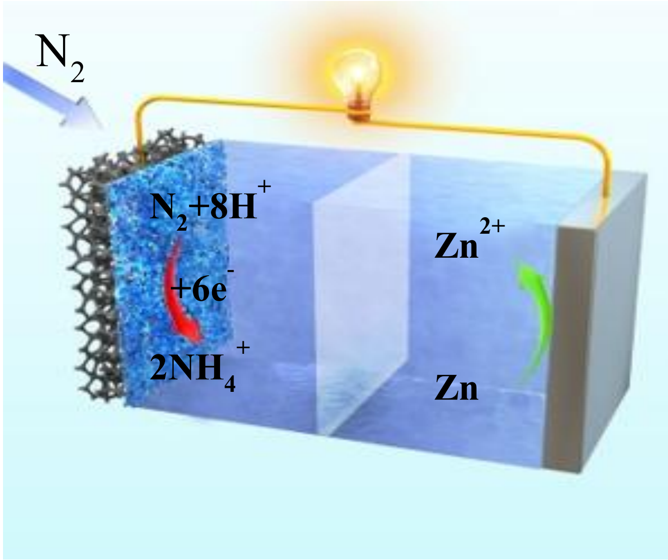

Figure S42. Schematic diagram of a rechargeable Zn-N_2 battery.

Table S6. Comparison of the Zn-N_2 battery performances of other electrocatalysts with $Co_xNi_{3-x}(HITP)_2$/BNSs.

**Catalysts**	**Power density (mW cm^{-2})**	**Energy density (mA h g^{-1})**	**Refs.**
exfoliated NbS_2	0.31	714	[17]
CoPi/NPCS	0.49	147.6	[18]
CoPi/HSNPC	0.31	138.6	[19]
Cu-2	0.01	-	[20]
$Fe_{1.0}$HTNs	0.028	6.51	[21]
VN@NSC	0.0164	-	[22]
$Co_xNi_{3-x}(HITP)_2$/BNSs	2.5	240.0	**this work**

References

[1] Y. Song, M. Xu, X. Liu, Z. Li, C. Wang, Q. Jia, Z. Zhang, M. Du, *Electrochim. Acta* **2021**, *368*, 137609.
[2] B. Delley, *J. Chem. Phys* **2000**, *113*, 7756-7764.
[3] J. P. Perdew, K. Burke, M. Ernzerhof, *Phys. Rev. Lett.* **1996**, *77*, 3865.
[4] B. Delley, *J. Chem. Phys.* **1990**, *92*, 508-517.
[5] D. Xing, Y. Wang, P. Zhou, Y. Liu, Z. Wang, P. Wang, Z. Zheng, H. Cheng, Y. Dai, B. Huang, *Appl. Catal. B-Environ.* **2020**, *278*, 119295.
[6] C. Hou, G. Tai, J. Hao, L. Sheng, B. Liu, Z. Wu, *Angew. Chem. Int. Ed.* **2020**, *59*, 10819-10825.
[7] H. Qiao, J. Yu, J. Lu, H. Bai, H. Liu, J. Hu, H. Huang, B. Wen, *ACS Sustain. Chem. Eng.* **2021**, *9*, 1373-1382.
[8] S. Chen, H. Jang, J. Wang, Q. Qin, X. Liu, J. Cho, *J. Mater. Chem. A* **2020**, *8*, 2099-2104.
[9] H. Y. Zhou, Y. B. Qu, J. C. Li, Z. L. Wang, C. C. Yang, Q. Jiang, *Appl. Catal. B-Environ.* **2022**, *305*, 121023.
[10] W.-B. Qiu, Y.-X. Luo, R.-P. Liang, J.-D. Qiu, X.-H. Xia, *Chem. Commun.* **2019**, *55*, 7406-7409.
[11] L. Han, X. Liu, J. Chen, R. Lin, H. Liu, F. Lü, S. Bak, Z. Liang, S. Zhao, E. Stavitski, J. Luo, R. R. Adzic, H. L. Xin, *Angew. Chem. Int. Ed.* **2019**, *58*, 2321-2325.
[12] Y. Zhang, J. Hu, C. Zhang, Y. Liu, M. Xu, Y. Xue, L. Liu, M. K. Leung, *J. Mater. Chem. A* **2020**, *8*, 9091-9098.
[13] L. Zhang, L.-X. Ding, G.-F. Chen, X. Yang, H. Wang, *Angew. Chem. Int. Ed.* **2019**, *58*, 2612-2616.
[14] A. Afif, N. Radenahmad, Q. Cheok, S. Shams, J. H. Kim, A. K. Azad, *Renew. Sust. Energ. Rev.* **2016**, *60*, 822-835.
[15] K. Chu, Y. Luo, P. Shen, X. Li, Q. Li, Y. Guo, *Adv. Energy Mater.* **2022**, *12*, 2103022.
[16] H. Zhong, M. Wang, M. Ghorbani-Asl, J. Zhang, K. H. Ly, Z. Liao, G. Chen, Y. Wei, B. P. Biswal, E. Zschech, *J. Am. Chem. Soc.* **2021**, *143*, 19992-20000.
[17] H. Wang, J. Si, T. Zhang, Y. Li, B. Yang, Z. Li, J. Chen, Z. Wen, C. Yuan, L. Lei, *Appl. Catal. B-Environ.* **2020**, *270*, 118892.
[18] J.-T. Ren, L. Chen, H.-Y. Wang, Z.-Y. Yuan, *ACS Appl. Mater. Inter.* **2021**, *13*, 12106-12117.
[19] J.-T. Ren, L. Chen, Y. Liu, Z.-Y. Yuan, *J. Mater. Chem. A* **2021**, *9*, 11370-11380.
[20] C. Du, Y. Gao, J. Wang, W. Chen, *Chem. Commun.* **2019**, *55*, 12801-12804.
[21] X.-W. Lv, X.-L. Liu, L.-J. Gao, Y.-P. Liu, Z.-Y. Yuan, *J. Mater. Chem. A* **2021**, *9*, 4026-4035.
[22] X.-W. Lv, Y. Liu, Y.-S. Wang, X.-L. Liu, Z.-Y. Yuan, *Appl. Catal. B-Environ.* **2021**, *280*, 119434.
